# Watching the Interplay between Photoinduced Ultrafast Charge Dynamics and Nuclear Vibrations

**DOI:** 10.1021/acs.jctc.3c00855

**Published:** 2023-11-22

**Authors:** Edoardo Buttarazzi, Fulvio Perrella, Nadia Rega, Alessio Petrone

**Affiliations:** † Scuola Superiore Meridionale, Largo San Marcellino 10, I-80138 Napoli, Italy; ‡ Department of Chemical Sciences, University of Napoli Federico II, Complesso Universitario di Monte S. Angelo, Via Cintia 21, I-80126 Napoli, Italy; § Istituto Nazionale Di Fisica Nucleare, sezione di Napoli, Complesso Universitario di Monte S. Angelo ed. 6, Via Cintia, I-80126 Napoli, Italy

## Abstract

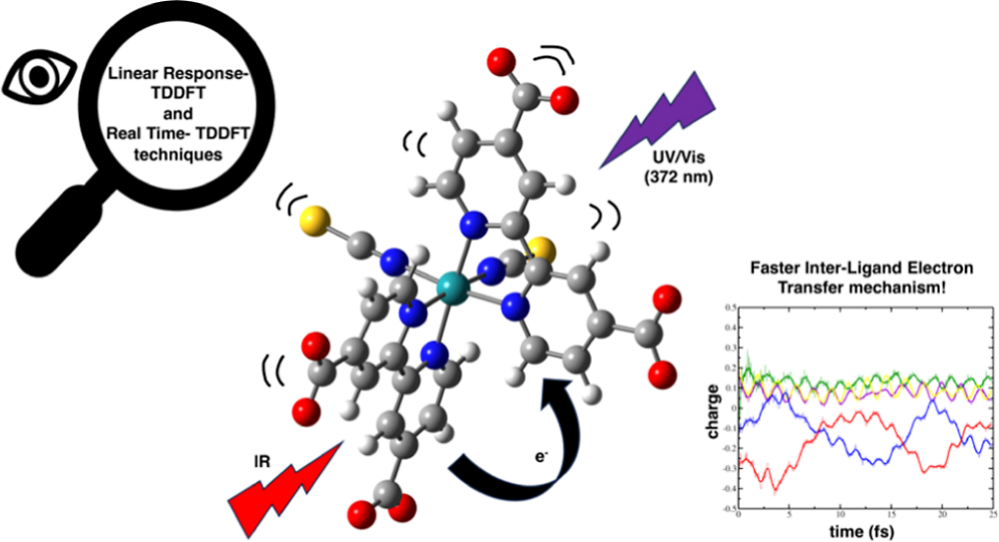

Here is presented the ultrafast hole–electron dynamics of photoinduced metal to ligand charge-transfer (MLCT) states in a Ru(II) complex, [Ru(dcbpy)_2_(NCS)_2_]^4–^ (dcbpy = 4,4′-dicarboxy-2,2′-bipyridine), a photoactive molecule employed in dye sensitized solar cells. Via cutting-edge computational techniques, a tailored computational protocol is here presented and developed to provide a detailed analysis of the electronic manifold coupled with nuclear vibrations to better understand the nonradiative pathways and the resulting overall dye performances in light-harvesting processes (electron injection). Thus, the effects of different vibrational modes were investigated on both the electronic levels and charge transfer dynamics through a theoretical-computational approach. First, the linear response time-dependent density functional (LR-TDDFT) formalism was employed to characterize excitation energies and spacing among electronic levels (the electronic layouts). Then, to understand the ultrafast (femtosecond) charge dynamics on the molecular scale, we relied on the nonperturbative mean-field quantum electronic dynamics via real-time (RT-) TDDFT. Three vibrational modes were selected, representative for collective nuclear movements that can have a significant influence on the electronic structure: two involving NCS^–^ ligands and one involving dcbpy ligands. As main results, we observed that such MLCT states, under vibrational distortions, are strongly affected and a faster interligand electron transfer mechanism is observed along with an increasing MLCT character of the adiabatic electronic states approaching closer in energy due to the vibrations. Such findings can help both in providing a molecular picture of multidimensional vibro-electronic spectroscopic techniques, used to characterize ultrafast coherent and noncoherent dynamics of complex systems, and to improve dye performances with particular attention to the study of energy or charge transport processes and vibronic couplings.

## 1 Introduction

Metal-to-ligand charge-transfer (MLCT) states are essential for photoexcited energy transfer steps in many natural and artificial light-harvesting processes and photocatalysis.^1–11^ The interplay between the perturbed electronic density (i.e., a sudden excitation induced by light absorption) and nuclear motions (nuclear vibrations) determines the simplicity with which an excited state returns to the ground state and/or the charge-recombination takes place, affecting the efficiency of the light harvesting process. Some motions can, indeed, lead to quenching, while others might induce faster charge injection, thus limiting/enhancing the efficiency of dyes for technological applications. More generally, to harvest the energy of a photogenerated electron–hole pair in such photoactive materials, it is necessary to separate the exciton (a Coulombic bound state of an electron and hole) and prevent it from recombining before the charges can reach the electrodes. In this context, the synergy between advanced spectroscopic techniques (time-resolved and nonlinear)^12–16^ and computational strategy can give the necessary tools to accurately understand the temporal evolution of the electronic density and the relative structure–function interplay.^17–26^ Moreover, multidimensional spectroscopic techniques, such as two-dimensional (2D) spectroscopy, i.e., 2D electronic spectroscopy (2DES)^27–34^ and hybrid 2D vibrational-electronic (2DVE),^35–39^ have already been successfully used to characterize ultrafast coherent and noncoherent dynamics of complex systems, with particular attention to the study of energy or charge transport processes and vibronic couplings. Research interests are being directed at understanding the role of coupled vibrations solvent dynamics, spin–orbit coupling, and the available excited density of states in determining the ultrafast photochemistry of transition-metal complexes.^36,40–59^

**Figure 1:**
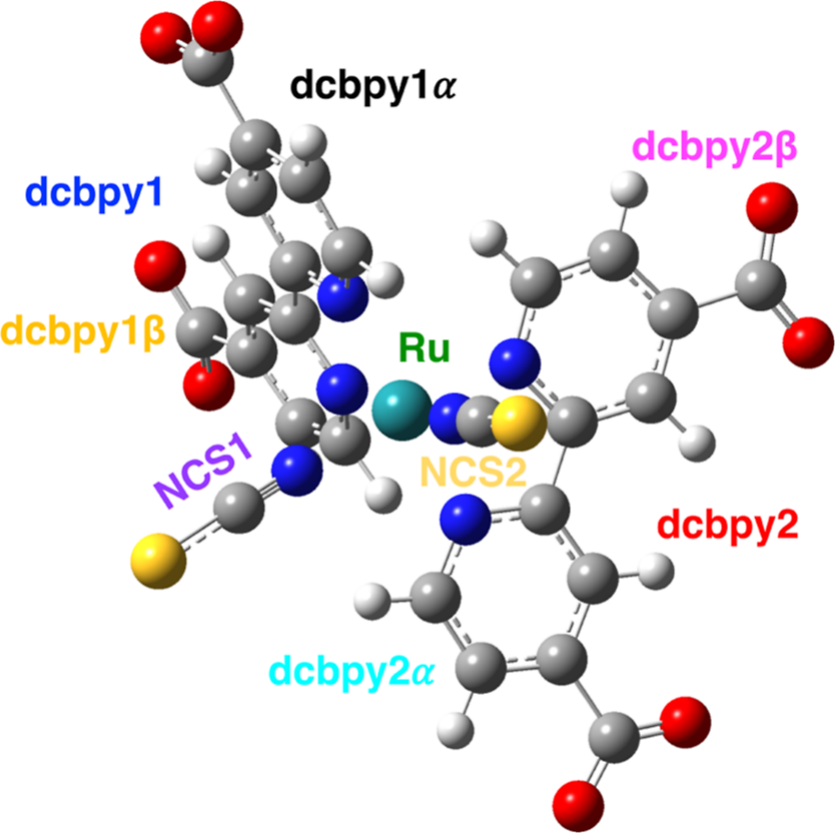
Optimized structure of the N3^4–^ metal complex in the gas phase. Fragments are labeled. Chosen level of theory is reported on the Computational Methods and Details section. Atoms color palette: C-dark gray; H-white; N-blue; O-red; S-yellow; Ru-turquoise.

Here, we present the ultrafast hole–electron dynamics of two experimentally relevant photoinduced charge-transfer excited states (belonging to the 372 nm band)^36^ of a Ru(II) complex, [Ru(dcbpy)_2_(NCS)_2_]^4–^ (dcbpy = 4,4′-dicarboxy-2,2′-bipyridine), also called “N3^4–^” (see Figure 1). The system was characterized and propagated, on the subfemtosecond time scale, to analyze the interplay of the dense electronic manifold of the metal complex with nuclear motions, substantial impacting on the CT dynamics and also on its interligand electron transfer (ILET) mechanism. N3, and its charged variants, belongs to a broader class of transition-metal compounds undergoing rapid and complex charge transfer dynamics, potentially influenced by structural rearrangements, and vastly employed as dyes in dye-sensitized solar cells.^41,60–62^ The complex photoinduced dynamics registered for N3 suggests that many states in the excited singlet ^1^MLCT manifold might be involved in various CTs and relaxation pathways available to these systems. Previous experimental works already suggested that ILET processes can occur in the Franck–Condon region of the singlet ^1^MLCT excited state manifold, i.e., before the intersystem crossing toward the final lowest energy triplet ^3^MLCT state, for the extensively studied Ru(bpy)_3_^2+^,^60,63–69^ as well as for N3^4–^.^70^ Recently, we have observed an ultrafast (<25 fs) time for ILET to occur,^24^ and additionally, some of the authors have been involved in the modeling of recent two-dimensional electronic-vibrational spectroscopy experiments that proved the crucial importance of vibrational modes affecting either both the Ru-(NCS) charge-donor segment or the dcbpy charge-acceptor portion on the N3^4–^ molecule, having first evidence of their correlation with many ^1^MLCT states and the excited state CT processes.^36^ Such states, called ^1^MLCT_*A*_ and ^1^MLCT_*B*_ and, respectively, found around 24 400–24 700 cm^–1^ (410–405 nm) and 25 000–25 300 cm^–1^ (400–395 nm), were previously shown to be those most vibronically coupled with the final (and energetically lowest) triplet ^3^MLCT state (main responsible, along with the initial singlet states, of the electron injection in the semiconductor in the photodevices). Both ^1^MLCT_*A*_ and ^1^MLCT_*B*_ states are characterized by an important electronic density redistribution from the Ru(NCS)_2_ moiety toward the dcbpy rings, with a transition electric dipole moment predominantly located on the N3^4–^ equatorial plane. Several studies^4,6,7,40,71–74^ proved that vibrational modes localized to the charge-donor and charge-acceptor can be coupled to the hole–electron and, more in general, CT dynamics, but a molecular investigation on the ultrafast time-scale of the interplay between the dense electronic manifold and such nuclear motions is not yet fully available. The interconnected contribution of nuclear vibrations and the electronic energy level displacement, the interplay among energetically close singlet states (before decaying into the final triplet state), and the time scales of interligand electron transfer are all still far to be understood, although pivotal to the light-harvesting efficiency of such dyes.

Exact electron–nuclear dynamics can be achievable by solving the full time-dependent Schrödinger equation for the entire (electronic plus nuclear) system. This approach is extremely accurate, but computationally prohibitive and affordable only for all but the smallest molecules with only a few active electrons.^75,76^ Several approximate methods have been introduced with the aim of achieving reliable results at lower costs,^77–93^ where two of the most widely used methods are trajectory surface hopping and the Ehrenfest methodologies. However, in this work we propose a detailed analysis of the electronic manifold coupled with nuclear vibrations for large molecules where the effects of different vibrational modes were investigated on both the electronic levels and ^1^MLCT_*A*_ and ^1^MLCT_*B*_ states dynamics of N3^4–^ via cutting-edge computational techniques and a tailored computational protocol here developed. To this aim, we first exploit the linear response time-dependent density functional (LR-TDDFT) formalism to characterize excitation energies and spacing among electronic levels (the electronic layout). Then, to understand the ultrafast (subpicosecond) charge dynamics on the molecular scale, we rely on the nonperturbative mean-field quantum electronic dynamics (ED) via real-time (RT-) TDDFT. RT-TDDFT has been vastly employed in the past to model charge transfer and excitation dynamics directly and precisely in several donor–acceptor systems^17,23,94–97^ and to provide the molecular interpretation of the interaction between initial photoexcited states,^22,98–106^ exciton and polaron formation,^107–110^ including relativistic effects.^111,112^ We refer readers to previous review publications^21,113,114^ for more detailed discussion on the subject.

The charge dynamics was analyzed along the vibrational distortion of three modes representative for collective nuclear movements that can have a significant influence on the electronic structure: two involving NCS^–^ ligands and one involving dcbpy ligands. We observed that the energetic layout of electronic states in proximity of such ^1^MLCT states, under vibrational distortions, is strongly affected, and a faster interligand electron transfer mechanism is observed. An increasing MLCT character of the adiabatic electronic states approaching closer in energy due to the vibrations is also clear. Such results can help both in providing a molecular picture of multidimensional vibro-electronic spectroscopic techniques, used to characterize ultrafast coherent and noncoherent dynamics of complex systems, and to improve dyes performances with particular attention to the study of energy or charge transport processes and vibronic couplings.

## 2 Computational Methods and Details

### 2.1 Energy Scan Profiles

**Figure 2:**
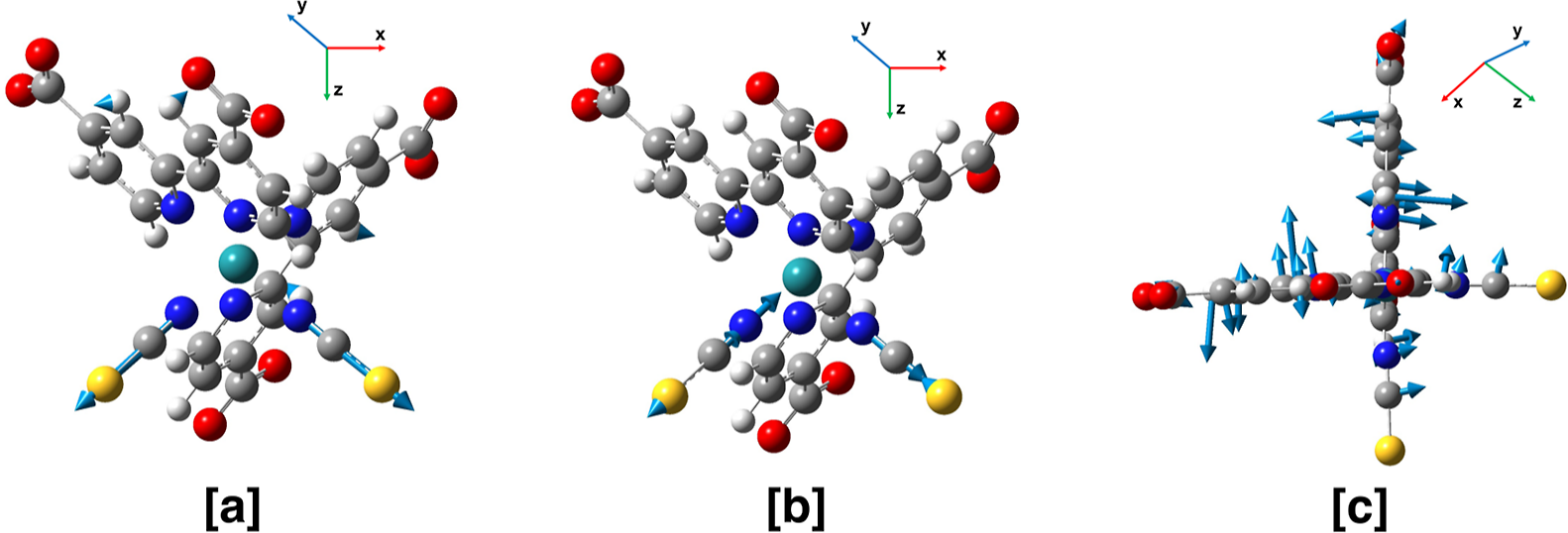
Selected vibrational modes and their vibrational displacement vectors. From left: **[a]**, **[b]**, and **[c]** modes. See the Results and Discussion section for a more detailed discussion.

Energy scan profiles of higher energetic electronic states (from S_17_ to S_27_, describing the experimentally relevant 372 nm band) along displacements of significant normal modes were computed. We first performed the harmonic normal-mode analysis on the ground state N3^4–^ minimum energy structure, then we selected three vibrational modes (hereafter named: **[a]**, **[b]**, **[c]**), involving the symmetric and asymmetric stretching motions of NCS^–^ and an out of plane motion of the dcbpy ligands, respectively (see Figure 2 and the Results and Discussion section for more details about this choice).

Displacement ranges along such modes were then selected on the basis of values adopted by significant structural parameters in a previously collected ab initio molecular dynamics simulation of the metal complex at room temperature.^24,115^ Furthermore, in detail, we considered the Ru–N(NCS), N(NCS)–N(dcbpy) distances and the Ru–N(NCS)–N(dcbpy) angle, since these structural parameters were the most affected by **[a]**, **[b]**, and **[c]** modes, and analyzed distributions of such parameters (minimum, maximum and standard deviation σ) during the trajectory. On the basis of such values, we could make a choice of the mode displacements well representative of the actual motion of the complex at room temperature. For vibrations **[a]**, **[b]**, **[c]**, normal mode displacements were selected in the ranges of ±0.025 (displacement resolution of 0.005), ±0.025 (displacement resolution of 0.005), and ±0.4 (displacement resolution of 0.02), respectively (see also Tables S1 to S5). On the resulting structures displaced along such modes, the vertical excitation energies from the ground to the first 40 singlet electronic excited states were computed through LR-TDDFT.^116–119^ Then, the energy scan profiles were plotted for 10 electronic states in the energetic range of interest of ^1^MLCT_*A*_ and ^1^MLCT_*B*_ states (from S_17_ to S_27_) in each vibrational mode case study. The energy profiles  were obtained as the electronic energy of the S_*n*_ state at each displacement relative to the ground state energy evaluated at the minimum energy structure through eq 1
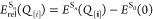
1where  is the energy of the S_*n*_ electronic state evaluated at each structure displaced from the minimum of an amount of *Q* along the **[i]** vibrational mode and *E*_0_^S^(0) is the energy of the symmetric and undistorted ground state minimum. *Q* is here a pure scalar number by which the Cartesian displacement vectors normalized to unit distance, obtained from the normal mode output, where multiplied, and where the zero value means no distortions from the minimum energy geometry.

Additionally, we also defined the energy difference between electronic states under particular vibrational distortions
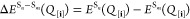
2

### 2.2 Real-Time TDDFT Charge Reorganization and MO Occupation Dynamics

The ultrafast electronic dynamics resembling the N3^4–^ photoinduced ^1^MLCT excited states was characterized and collected. ED were performed on different nuclear configurations, obtained through the distortion of the N3^4–^ ground state minimum structure along three vibrational modes (see Tables S1 to S5 and the previous paragraph). Once the nuclear configurations were fixed, we focused on the ultrafast electronic reorganization after photoexcitation. In these early stages of charge dynamics (∼25 fs), we can neglect the direct couplings of nuclear motions on the charge reorganization,^24,120^ since the motions here analyzed are not ultrafast, given that from their computed harmonic frequencies (see Results and Discussion section) we can see that **[a]** and **[b]** have period of about ∼40 fs and **[c]** is even slower, with a period of about ∼105 fs. This computational experiment can provide the explicit evolution of the electronic density in time, mathematically represented in terms of time evolving orbital coefficients or of the one-electron density matrix **P** elements on an orthonormal atom centered basis. The time evolution of a specified initial photoexcitation is resolved through RT-TDDFT calculations, in which the electronic density matrix is propagated in time according to the nonlinear Liouville–von Neumann equation^121,122^
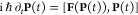
3where i is the imaginary unit, *ℏ* is the reduced Planck constant, ∂_*t*_ is the partial derivative with respect to time, and **F** is the Fock (Kohn–Sham within DFT framework) matrix in the orthonormal basis. Formally, eq 3 may be propagated in the time domain (given an initial time *t*_0_) through the Magnus expansion of the time-domain propagator^123^
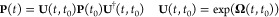
4where the † symbol is the Hermitian conjugate and **Ω**(*t*, *t*_0_) is a nonterminating series expansion which is to be elided. As integration scheme, we adopted the modified midpoint unitary transformation, MMUT, method.^101^ MMUT is a symplectic multistep (leapfrog) explicit integration scheme based on the Magnus expansion with error formally Δ*t*^2^ (*t*_*k*_ is the current time step during the temporal propagation)
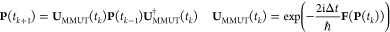
5

The easiest procedure for preparing an initial state resembling an electronic excitation is to directly adjust the orbital populations without relaxation by promoting an electron to an unoccupied orbital^107^ (we refer the reader to refs (124–127) for detailed overviews about additional and alternative approaches proposed over the years). Thus, in order to obtain the initial electronic perturbation to perform electronic dynamics, the excited states of interest are prepared by promoting an electron from a selected occupied molecular orbital to one that is unoccupied in the ground state (Koopmans excitation) according to the electronic transition of interest between the singlet ground state minimum (S_0_) and the nth singlet excited state (S_*n*_), whose main orbital contributions are resolved by using preliminary frequency domain LR-TDDFT calculations. The Koopmans excitation step creates a nonstationary electron density that is representative of a coherent superposition of the ground and excited states of interest, according to a well-established procedure.^20,21,23,110,128,129^ Comparison of spatial and electric dipole features allowed us to associate the S_*n*_ states involved in the S_0_ → S_*n*_ transitions with the ^1^MLCT state, responsible for the ultrafast dynamics observed experimentally in the 2D electronic-vibrational spectra.^36^

To expose the most relevant features of the electron dynamics of the chosen metal complex N3^4–^, several parameters were evaluated along the RT-TDDFT trajectories in the time domain. Time-dependent properties were extracted for this aim from the time-evolving density. To provide a spatial representation of the CT dynamics, the occupation of molecular orbitals, n_*i*_(*t*), was followed in terms of time
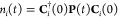
6where the time-evolving electronic density matrix is projected onto the ground state molecular orbitals (MOs) basis, represented as a linear combination of atomic orbital basis at zero time, **C**(0), and *n*_*i*_(*t*) is the occupation number of the |ψ_*i*_(0)⟩ ground state MO. The occupation of several frontier orbitals was tracked for the entire ED (from HOMO–20 to LUMO+20).

### 2.3 Simulation Details

The global hybrid Becke, 3-parameter, Lee–Yang–Parr (B3LYP) density functional was chosen^130–132^ in combination with def2SVP for all the atoms (H, N, C, O, S) with the def2SVP basis set and associated electronic core potential (ECP) for Ru.^133^ Solvent effects were not taken into account in the current study, and all calculations were performed in the gas phase. This level of theory was already validated in previous studies for the system under investigation.^24,36,115,120,134^ Vertical excitation energies and corresponding oscillator strengths were calculated using the LR-TDDFT.^118,119,135^ Electron dynamics trajectories were simulated within RT-TDDFT framework using a developer version of the Gaussian suite of programs.^136^ ED trajectories were propagated for 25 fs each, with a time step of 1 as. Mulliken population analysis^137,138^ was employed to obtain time-dependent fragment charges along RT-TDDFT trajectories every 50 as. All calculations, beside RT-TDDFT ED, were performed using the commercial electronic structure software suite Gaussian 16, C01 version.^139^

## 3 Results and Discussion

### 3.1 Electronic State Layout on Ground State Minimum Energy Structure

**Figure 3:**
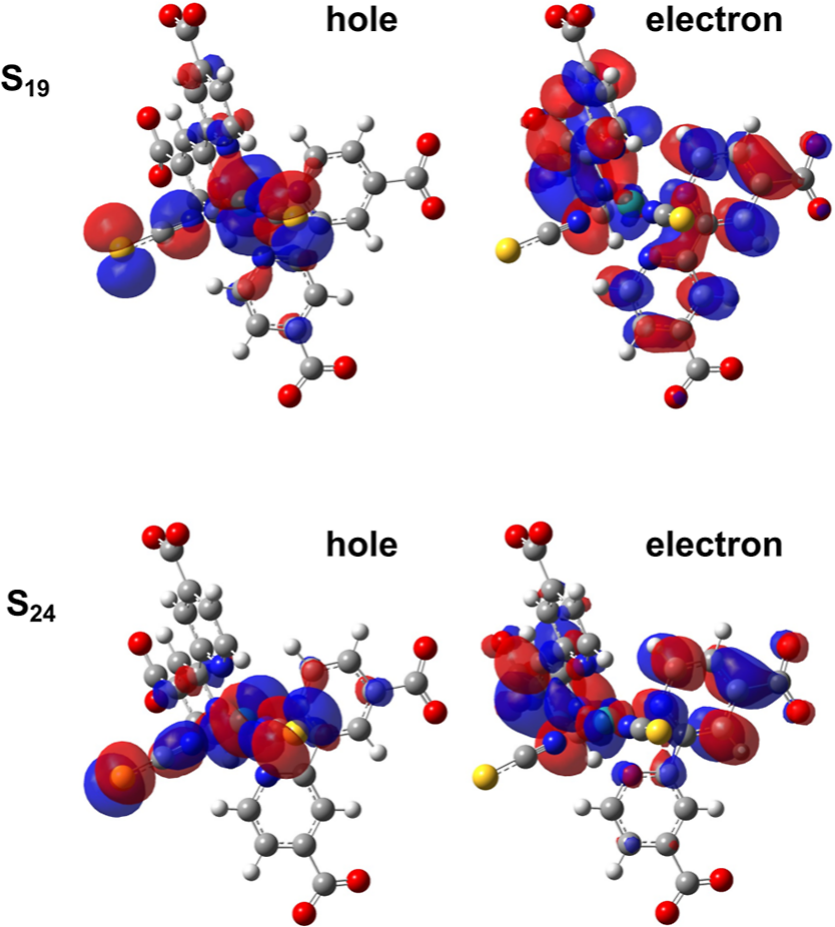
NTOs of hole and electron for the electronic transitions toward the S_19_, resembling the ^1^MLCT_*A*_ character and to S_24_, resembling the ^1^MLCT_*B*_ character for N3^4–^ ground state minimum. Isovalue: 0.02. See the text for their labelings and definitions.

The nature of ^1^MLCT_*A*_ and ^1^MLCT_*B*_ states have been extensively studied in the past for this complex by some of the Authors.^24,36,120^ In such works, it was experimentally measured that the difference between the highest and lowest excitation energies to be associated with the corresponding adiabatic states, S_*n*_ should be ∼450–650 cm^–1^, as observed in the peak separation for excitations into ^1^MLCT_*A*_ and ^1^MLCT_*B*_. A correlation between the located adiabatic states and electric dipole was also conducted to better understand the identification of corresponding adiabatic states. The adiabatic states relative to the ground state minimum energy structure and corresponding to the ^1^MLCT_*A*_ and ^1^MLCT_*B*_ were found to be the transitions toward to S_19_ and S_24_ adiabatic states, respectively (see also refs (24),^120^ for a detailed discussion on such states). We first report in Table 1 the vertical excitation energies from the electronic ground state to the selected higher energy states of interest (from S_17_ to S_27_) relative to the ground-state energy minimum N3^4–^ metal complex structure. The adiabatic electronic state, corresponding to the ^1^MLCT_*A*_ has two immediately preceding states (S_18_ and S_17_) and one immediately following state (S_20_) within 0.02 eV of energy. Also, the adiabatic electronic state corresponding to the ^1^MLCT_*B*_ has several energetically close states (S_20_, S_21,_ S_22_, S_23_ and S_25_, S_26_, respectively) within ∼0.05 eV of energy. This behavior shows that the system presents a very dense electronic manifold in the proximity of such MLCT states, although MO pairs involved are not always similar (see last column of Table 1). In fact, S_19_ mainly originated from the HOMO–1 to LUMO+2 transition and S_24_ from the HOMO to LUMO+5 transition (see Figure S1 for MO representations). These states also contain contributions from transitions between other MO pairs (Table 1). Then, the nature of the hole and electron, via natural transition orbital (NTO),^140^ was analyzed to describe the characteristics of such electronic states with large MLCT character to be used later on to follow their evolution along the analyzed mode as well. In Figure 3 contours of the hole and electron NTOs of both the S_19_ and S_24_ transitions are reported. The hole NTOs for both S_19_ and S_24_ transitions involve the d orbitals of the Ru atom and NCS^–^ ligands (NCS1 and NCS2 in Figure 1). On the other hand, dcbpy ligand aromatic rings are involved in the electron NTO for both S_19_ and S_24_ excitations. In particular, the S_19_ electron NTO includes both rings (α and β fragments in Figure 1) in dcbpy ligands with a symmetrical layout (Figure 3), while the S_24_ electron NTO is mostly localized on the β portion (please refer to Figure 1 for the fragment definition) of the dcbpy ligand. We have also analyzed the nature of electronic transitions toward the states in the higher energetic electronic manifold (S_17_–S_27_), the corresponding NTOs are reported in Figures S2 and S3. Transitions toward S_17_, S_22_, S_23_, S_25_, and S_26_ consist in an intraligand electron transfer from the carboxylate groups of dcbpy ligands (hole NTOs) to the aromatic portion of dcbpy ligands (electron NTOs). Such transitions mostly differ for the different involvement of the carboxylate groups in the hole NTOs (α portions for S_17_, S_25_, and S_26_; β portions for S_22_ and S_23_) and/or for the different symmetry of the density of the aromatic portion of dcbpy in electron NTOs. S18 and S20 electronic transitions have a mixed MLCT character, combining a charge transfer from NCS-Ru to the accepting dcbpy ligands with also a partial ligand (NCS) to ligand (dcbpy) charge transfer contribution (S_18_ and S_20_ invert their hole and hole-1 NTOs), see Figure S2. S_21_ has still an MLCT character, similar to S_19_, but we assist to a different symmetry of the involved d orbital in the hole NTO for the metal center. S_27_ electronic transition is similar to S_24_, still having MLCT character, but this time the electron NTO is more localized on the α rings of dcbpy ligands (rather than the β portion as in S_24_).

**Table 1:** LR-TD-B3LYP Vertical Excitation Energies [Δ*E*^S_n_–S_0_^(0)] for the N3^4–^ Ground State Energy Minimum Structure^a^

S_*n*_	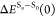	MO pairs	*f*
S_17_	3.038	h–11 → l (0.28); h–10 → l+1 (0.20)	0.000
S_18_	3.050	h–7 → l+1 (0.19); h–2 → l+2 (0.01); h → l+5 (0.28)	0.008
S_19_	3.058	h–1 → l+2 (0.43); h → l+5 (0.06)	0.001
S_20_	3.079	h–7 → l+1 (0.30); h–2 → l+2 (0.03); h → l+4 (0.16)	0.036
S_21_	3.118	h–2 → l+2 (0.44); h–1 → l+3 (0.01); h → l+4 (0.03)	0.023
S_22_	3.124	h–4 → l+1 (0.33); h–3 → l (0.17)	0.000
S_23_	3.124	h–4 → l (0.17); h–3 → l+1 (0.33)	0.000
S_24_	3.152	h–16 → l+1 (0.01); h–2 → l+1 (0.01); h–2 → l+5 (0.02); h–1 → l+2 (0.04); h–1 → l+4 (0.03); h → l+3 (0.02); h → l+5 (0.32)	0.045
S_25_	3.176	h–6 → l+1 (0.29); h–5 → l (0.21)	0.001
S_26_	3.176	h–6 → l (0.21); h–5 → l+1 (0.28)	0.001
S_27_	3.226	h–2 → l+4 (0.01); h–1 → l+3 (0.4); h–1 → l+5 (0.03)	0.036

a Energies are expressed in eV. MO pairs involved and their largest coefficients in the CI expansion according to the LR-TD-DFT formalism^116–119^ (in parentheses for each pair, as squared values) in the MO basis pairs are reported. In the last column are reported the oscillator strength values (*f*, dimensionless). h and l represent HOMO and LUMO, respectively.

### 3.2 Vibrational Effects on High Energy MLCT States and Electronic Manifold

**Figure 4:**
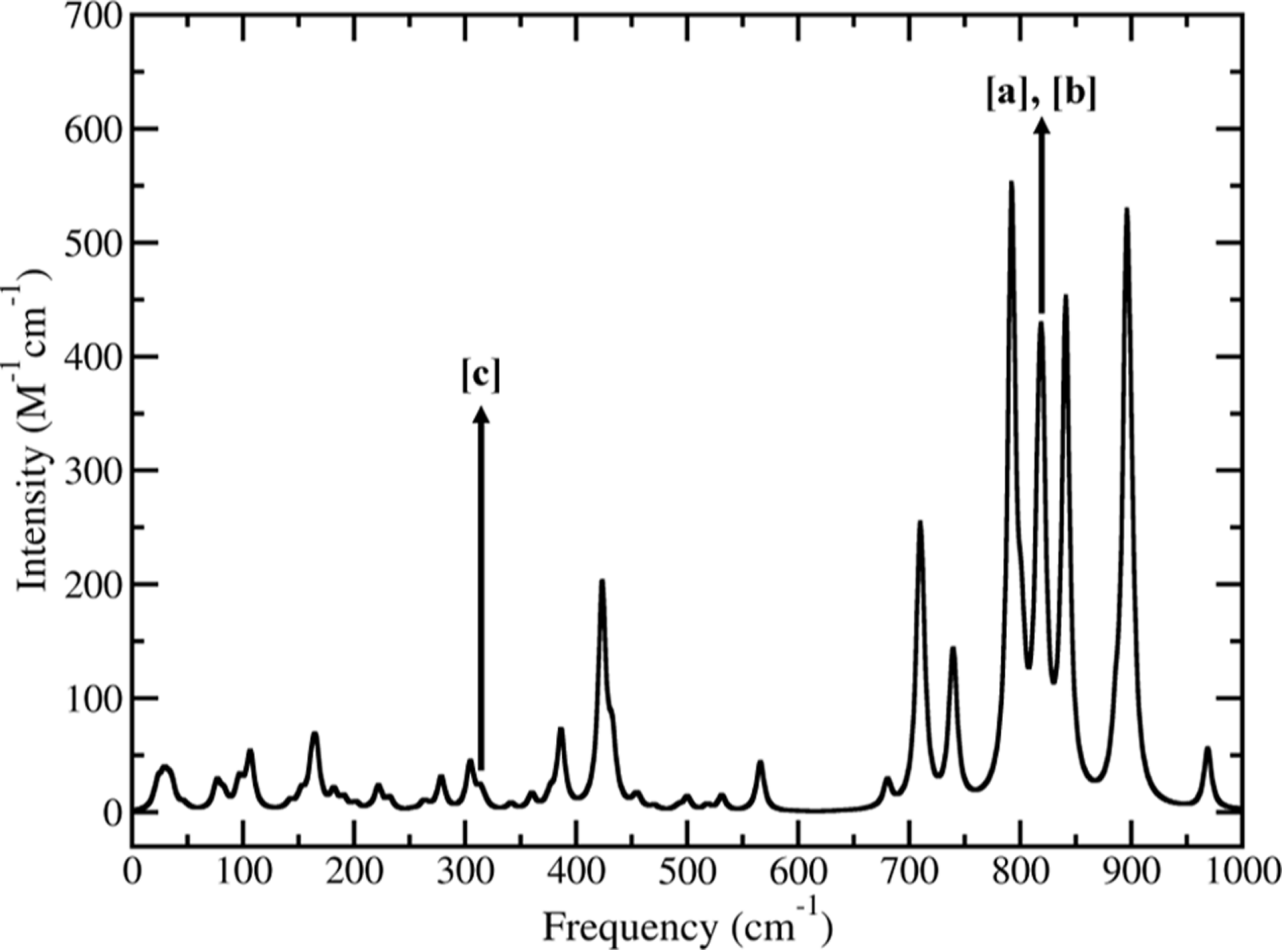
Computed harmonic infrared spectrum of N3^4–^. Vibrational modes of interest are labeled. Infrared peak width is 4 cm^–1^.

Once the harmonic normal-mode analysis on the ground state N3^4–^ minimum energy structure was performed, we focused on **[a]**, **[b]**, and **[c]** modes because they are representative for collective nuclear movements that can have a significant influence on the electronic structure. The computed infrared spectrum is reported in Figure 4. The level of theory here employed was already proved to be reliable for describing the nature of the vibrational motions (see ref (36)). Reproducing the IR spectrum is not the aim of this work since the current study is in the gas phase and a direct comparison with experiments cannot be easily made. On another hand, previous studies^49,141,142^ have unveiled that low-frequency vibrations affecting the Ru–N and Ru-dcbpy motions are also involved in potential energy crossings and ILET due to excited-state mixing and have stressed the importance to understand the role of the ligands in realizing long-lived charge separation and the interplay between the relevant electronic and vibrational coordinates. In addition, we focused on this frequency region since we are mostly interested in the normal modes that are more collective, more active also at lower temperatures, and then capable of affecting the overall geometries and the metal–ligand and ligand–ligand relative orientations and spatial distances. Such motions are also IR active, potentially excited at room temperature, and are among the best candidates to tune the overall electronic distribution and the electronic layout, i.e., by changing the energy separation among electronic states and their nature. Thus, for such reasons, we chose the following molecular vibrations (see Figure 2 for their representations in terms of normal mode displacement vectors): the symmetric (referred to as **[a]**) and asymmetric (referred to as **[b]**) stretching of NCS^–^ ligands (along the Ru–N direction) and collective/backbone modes of dcbpy ligands (referred to as **[c]**). This last vibration involves a synchronized motion of the two dcbpy ligands, making them closer (or farther) with respect to each other. Such vibrations can potentially influence the mechanism and the rate ruling a previously observed ILET among the dcbpy ligands.^24,120^ The computed harmonic frequencies for such vibrational modes are the following: **[a]** is 820.0 cm^–1^; **[b]** is 816.9 cm^–1^; and **[c]** is 313.6 cm^–1^.

**Figure 5:**
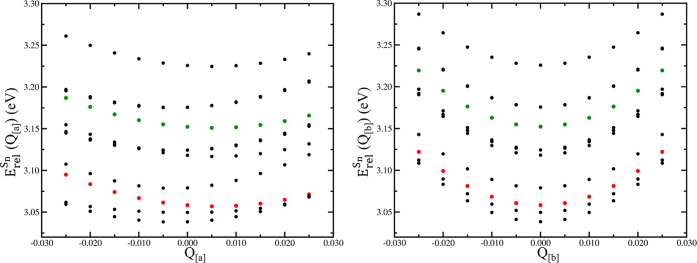
LR-TDB3LYP energy profiles of several electronic states (from S_17_ to S_27_) along the **[a]** (left panel) and **[b]** (right panel) vibrational modes. Energies are calculated as relative to the energy of the reference ground state minimum (eq 1). MLCT characters of the states of interest are colored (^1^MLCT_*A*_, red dots, and ^1^MLCT_*B*_, green dots).

Energy scans along such selected normal mode coordinates were performed (see the Computational Methods and Details section and Figure 2 for details). In particular, the vertical excitation energies of several electronic transitions from the ground to the previously analyzed higher energy electronic states of the N3^4–^ complex (from S_17_ to S_27_) were investigated under different vibrational displacements. Such analysis is useful to gauge the activation of dissipative channels induced by such vibrations. In the Figure 5 left panel, the energy profiles from S_17_ to S_27_ electronic states under vibrational mode **[a]** are shown. Vibration **[a]** is the symmetric stretching of NCS^–^ ligands along the Ru–N(NCS) direction (see Figure 2 as reference), and in the first place, we analyzed the variation of Ru–N(NCS) distance for definite displacement values (+0.025 and −0.025; see the Computational Methods and Details section) from the ground state minimum energy structure, used as reference. For +0.025 displacement, the Ru–N(NCS) distance is 2.056 against 2.066 Å of the minimum energy structure, and for −0.025 displacement, we noted an elongation of the aforementioned distance (2.077 vs 2.066 Å). For the other Ru–N(NCS) distance (the second NCS^–^ ligand), we observed the same behavior.

From the left panel of Figure 5, we observe that energy profiles are not symmetric along the *Q*_[*a*]_ value; this phenomenon is a direct consequence of the different variation of Ru–N(NCS) distance along the positive and negative displacements. For Ru–N(NCS) elongation (negative values of *Q*_**[a]**_), the adiabatic electronic state with the larger ^1^MLCT_*A*_ and ^1^MLCT_*B*_ character are well separated in energy from their immediately preceding electronic states, getting instead closer to those at immediately higher energy. On the other hand, for Ru–N(NCS) shortening (positive *Q*_**[a]**_ values), the adiabatic electronic states with the larger ^1^MLCT_*A*_ and ^1^MLCT_*B*_ character are well separated in energy from their immediately higher electronic states, getting instead closer to those immediately lower in energy. It is interesting to note how the **[a]** vibrational mode has a larger effect on destabilizing the energy of both HOMO–1 and HOMO (rather than affecting the energy of virtual MOs, see Walsh diagrams in Figures S5, S9 and S13, for all analyzed modes) involved the most in the hole NTOs of the states resembling ^1^MLCT_*A*_ and ^1^MLCT_*B*_, respectively. These MOs are d-orbitals of the Ru-center and π-orbitals of the NCS ligands with different symmetry (see their representations in Figure S5, highlighted with red and green picture frames, respectively). From our electronic dynamics (see next section), we can show that upon excitation the Ru has a larger positive charge (during our simulations the metal can be considered partially oxidized due to the electron transfer toward the dcbpy ligands), so the negative charged NCS ligands, getting closer to the metal center, are more able to stabilize the more positive charged metal center. It can also be inferred from the NTO analysis (see Figure 3) that upon excitation, the π NCS orbitals are also more available for retro-donation from the metal center, increasing the Ru–N(NCS) interaction. Thus, such MLCT states present energetic minimum geometries with a shorter Ru–N(NCS) distance (along the positive displacement in the graph) with respect to the ground state (as can be seen also by the Walsh diagram, where instead, the change of this distance destabilizes the occupied orbital into the ground state). In Table 2, we report the variation of energy (Δ  (Q_**[a]**_), see eq 2) for the adiabatic electronic states with the larger ^1^MLCT_*A*_ and ^1^MLCT_*B*_ character with respect to immediately preceding and following electronic states (S_18_ and S_20_ for ^1^MLCT_*A*_, and S_23_ and S_25_ for ^1^MLCT_*B*_), calculated at the equilibrium structure (*Q*_**[i]**_ = 0, second column) and at the chosen distortion (*Q*_**[a]**_ = ± 0.025, third and fourth columns, respectively). In both cases, we observe that the energy separation increases/decreases of ∼50%, indicating a potential larger mixing with such electronic states at the distorted geometries.

**Table 2:** LR-TD-B3LYP  (See eq 2) Energy Difference of the Adiabatic S_19_ and S_24_ against S_18_, S_20_, S_23_, and S_25_ Electronic States, Calculated at the Ground State Minimum (Second Column), and when Affected by **[a]** (Third and Fourth Columns), **[b]** (Fifth Column), and **[c]** (Sixth Column) Vibrations^a^

S_*n*_–S_*m*_	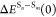	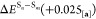	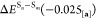	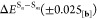	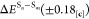
S_19_–S_18_	+0.009	+0.002	+0.033	+0.010	+0.002
S_19_–S_20_	–0.021	–0.048	–0.013	–0.021	–0.026
S_24_–S_23_	+0.028	+0.011	+0.032	+0.023	+0.007
S_24_–S_25_	–0.023	–0.040	–0.009	–0.026	–0.026

a Energies are expressed in eV.

Then, we studied the influence of vibrations on the aforementioned electronic states through NTO and MO analysis. NTO analysis has been performed to visualize the hole and electron of the adiabatic electronic state with larger ^1^MLCT_*A*_ and ^1^MLCT_*B*_ character in displaced structures with *Q*_**[a]**_ = ± 0.025. Comparing NTOs in Figure 3 (minimum energy structure) and in Figure S4 (displaced geometries along mode **[a]**), we can infer that vibrational mode **[a]**, at ±0.025 displacement values, does not alter the overall character of the MLCT states (see Figure 3 as reference).

Vibration **[b]** is the asymmetric stretching of NCS^–^ ligands along the Ru–N(NCS) direction (see Figure 2 as reference). Also, for **[b]**, we analyzed the variation of the Ru–N(NCS) distance along the displacements. For *Q*_**[b]**_ = +0.025 displacement, one of the Ru–N(NCS) distance is 2.055 Å, against the value of 2.066 Å in the ground state minimum energy structure; for *Q*_**[b]**_ = −0.025 we observe an elongation of the aforementioned distance (2.077 vs 2.066 Å). For the other NCS^–^ ligand, we detect an opposite behavior, given the asymmetric nature of the stretching mode. In the right panel of Figure 5, the energy profiles of S_17_ to S_27_ excited electronic states along the **[b]** vibrational distortion are reported. We note that the energy profiles along the **[b]** mode are symmetrical, regardless of the sign of the displacement. This is because while one NCS^–^ ligand is approaching the metal center, the other NCS^–^ ligand moves away from it. The energy differences of the adiabatic electronic state with the larger ^1^MLCT_*A*_ and ^1^MLCT_*B*_ character against S_18_, S_20_, S_23_, and S_25_ at *Q*_**[b]**_ = ± 0.025 were calculated and reported in the fifth column of Table 2. In this case, *Q*_**[b]**_ = ± 0.025 displacements do not affect the energy differences among the case study electronic states. In fact,  (±0.025_**[b]**_) energy values are closer to the ground state minimum ( (0)), at variance of vibration **[a]**. We analyzed NTOs in the +0.025 displaced structure and report them in Figure S8; **[b]** vibration affects mostly the symmetry of the π orbitals around the NCS^–^ ligands (^1^MLCT_*A*_ hole) resulting in a larger magnitude on the NCS^–^ closer to the metal center rather than the other ligand.

**Figure 6:**
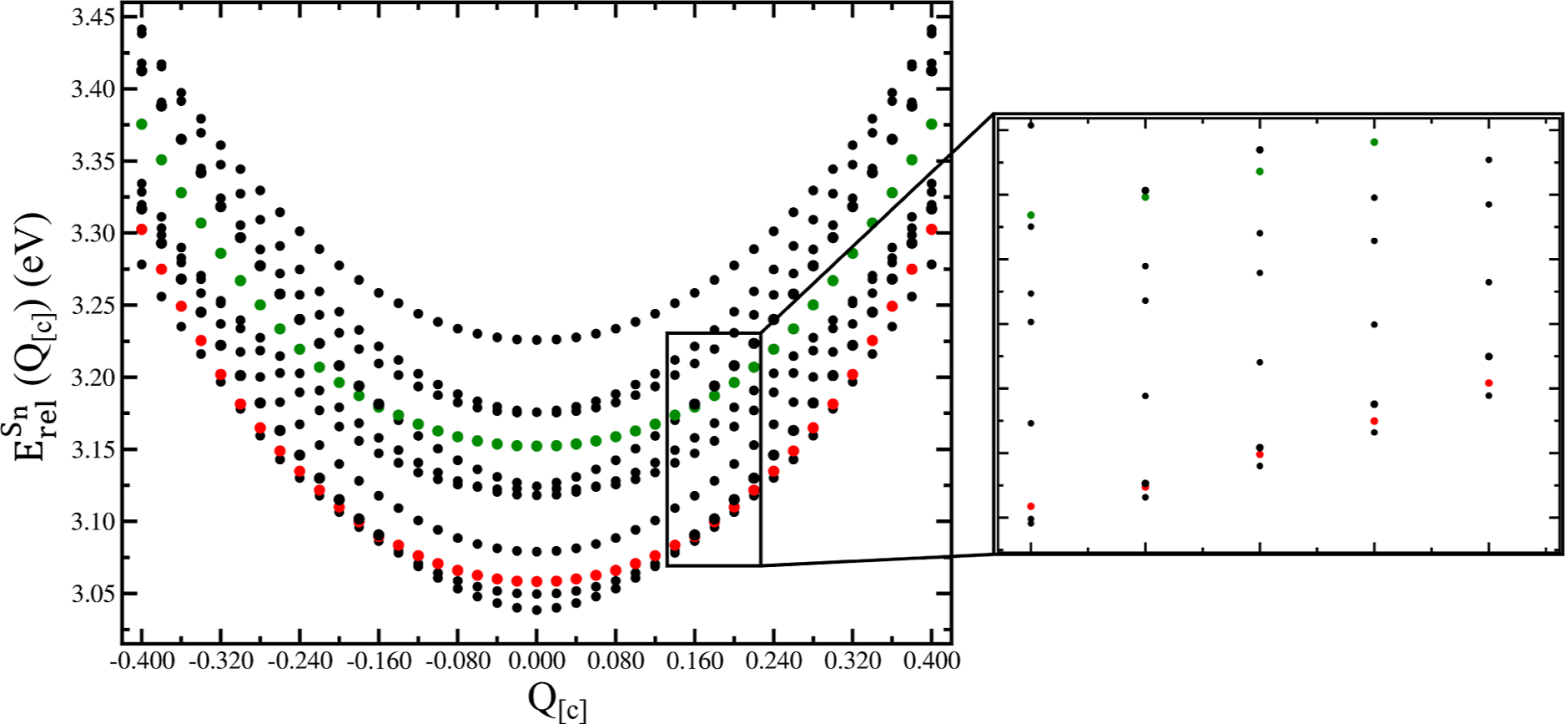
LR-TDB3LYP energy profiles of several electronic states (from S_17_ to S_27_) along vibrational mode **[c]**. Energies are calculated as relative to the energy of the undisplaced ground state minimum (eq 1). Magnified energy profiles from +0.14 to +0.22 displacement values are represented in the right panel. MLCT characters of states of interest are colored (^1^MLCT_*A*_, red dots, and ^1^MLCT_*B*_, green dots).

Finally, we analyzed vibration **[c]** that includes backbone and collective modes of dcbpy ligands. By a combined inspection of NTOs and energy scan profile analysis (see Figure 6), we found that the **[c]** effect on energy is symmetric regardless of the sign of the displacement. This occurs because **[c]** is a vibrational mode composed of twisting motions, whose center is on the dcbpy ligands. Through NTO analysis, we find that beyond a certain displacement value, the MLCT character of adiabatic states changes; for instance, S_18_ and S_23_ states along such coordinate are now the ones resembling the most the ^1^MLCT_*A*_ and ^1^MLCT_*B*_. In particular, beyond a displacement of *Q*_**[c]**_ = ± 0.14, the electronic state with the larger ^1^MLCT_*A*_ character swaps with the energetically preceding state (S_18_), while the electronic state with the larger ^1^MLCT_*B*_ character has already interchanged with the previous state (S_23_) at *Q*_**[c]**_ = ± 0.12.

We analyze the energy difference of the adiabatic electronic states with the larger ^1^MLCT_*A*_ and ^1^MLCT_*B*_ character against S_18_, S_20_, S_23_, and S_25_, also along the **[c]** mode, precisely at *Q*_**[c]**_ = ± 0.18 (see Table 2, last column). We chose *Q*_**[c]**_ = +0.18 because at this displacement we already previously observed the inversion of both the adiabatic electronic states with the larger ^1^MLCT_*A*_ and ^1^MLCT_*B*_ character with the immediately energetically preceding states. By inspecting Table 2, sixth column, displacement along the **[c]** mode results in a minor energy spacing for the S_19_, S_18_ and S_24_, S_23_ states with respect to the ground state minimum (S_24_–S_23_: +0.007 vs +0.028 eV). The same observations are valid also by comparison with respect to the previously analyzed displaced structures along the **[a]** and **[b]** modes. Thus, we observed that vibrational modes **[a]**, **[b]**, and **[c]** affect the energy and orbitalic contributions for such MLCT states. As a matter of fact, such distortions have non-negligible effects on the energetics of such adiabatic states resembling the most the MLCT states. We report in Figures S7, S11, and S15 the energetic gradients of these states along such normal coordinates. These results show that these modes are representative of collective nuclear movements that can have a significant influence on the electronic structure. The **[b]** mode seems to have a larger effect (but of the same order of magnitude of **[a]** and **[c]**) in the overall absolute change of the derivative. The **[c]** mode shows also a change in the slope that can help in understanding the different (diabatic) nature of the states along such coordinates and highlights the switch between adiabatic states that resemble the most the MLCT state at the chosen geometry. To understand how the examined nuclear vibrations can potentially influence the ultrafast charge dynamics, we chose to collect and analyze the ultrafast charge dynamics on the four following displaced structures: for **[a]**, *Q*_**[a]**_ = ± 0.025; for **[b]***Q*_**[b]**_ = +0.025 (symmetric energy profile) and for **[c]***Q*_**[c]**_ = +0.18 (symmetric energy profile).

### 3.3 RT-TDDFT Electronic Dynamics

The ultrafast charge reorganization observed for the undistorted minimum energy structure was in part previously presented by some of us in ref (24). We first summarize this charge reorganization in the minimum energy structure to have a reference for our investigation about the vibrational effects on the electronic dynamics for the initial densities resembling the excitations toward the (superposition of) electronic states with the larger ^1^MLCT_*A*_ and ^1^MLCT_*B*_ character of interest using the Koopmans excitation formalism explained in the Computational Methods and Details section. ^1^MLCT_*A*_ charge dynamics presents a positive charge difference vs the ground state that is shared among the metal center (Ru fragment at *t* = 0, +0.05 a.u.) and both NCS^–^ ligands (+0.21 a.u. for both NCS1 and NCS2 fragments at *t* = 0) for the entire evolution time of 25 fs, indicating the hole localization on these fragments. On the other hand, both dcbpy ligands have the same value of negative electronic charge difference (−0.24 a.u. for both dcbpy1 and dcbpy2 fragments at *t* = 0) and their charge dynamics is symmetrical and overlapped, indicating a shared localization of the electron. ^1^MLCT_*B*_ shows different dynamics with respect to ^1^MLCT_*A*_. Although NCS^–^ and dcbpy ligands charge dynamics are initially similar to ^1^MLCT_*A*_, after ∼15 fs we assist in an ILET process between the dcbpy ligands (i.e., symmetry breaking of the dcbpy charge dynamics) where the dcbpy2 fragment accumulates more negative charge difference than the dcbpy1 fragment. Due to the ILET process, symmetry of charge dynamics of NCS1 and NCS2 fragments is reduced. As previously noted in ref (24), for the ^1^MLCT_*B*_ state a clear ILET process is observed within ∼15 fs leading to a randomization of the electron among the ligands and with no nuclear distortion involved.

We now present the RT-TDDFT results for N3^4–^ along **[a]**, **[b]**, and **[c]** vibrational modes. For both **[a]**-distorted structures (*Q*_**[a]**_ = ± 0.025), we prepared the initial density, resembling the ^1^MLCT_*A*_ and ^1^MLCT_*B*_, performing the following MO swap: h–1 to l+2 and h to l+5, respectively (please see the Computational Methods and Details section for procedure and LR-TDDFT data in Table 1 and Figure S1 for MO choice).

**Figure 7:**
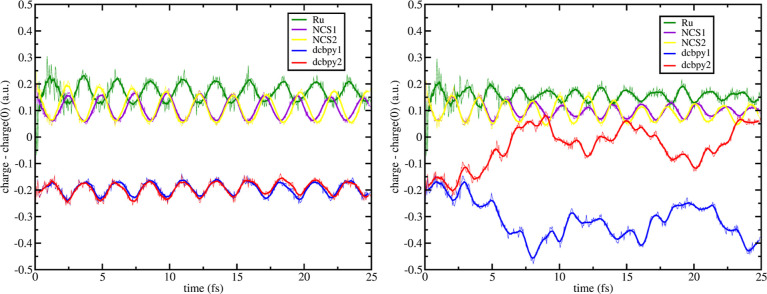
–0.025_**[a]**_ RT-TDB3LYP fragment charges difference dynamics (with respect to the S_0_ state) of states ^1^MLCT_*A*_ (left) and ^1^MLCT_*B*_ (right) for N3^4–^. Mulliken population analysis was performed every 50 as. Line smoothing was done through 10 steps window running averages. For fragment definitions see legend and Figure 1.

**Figure 8:**
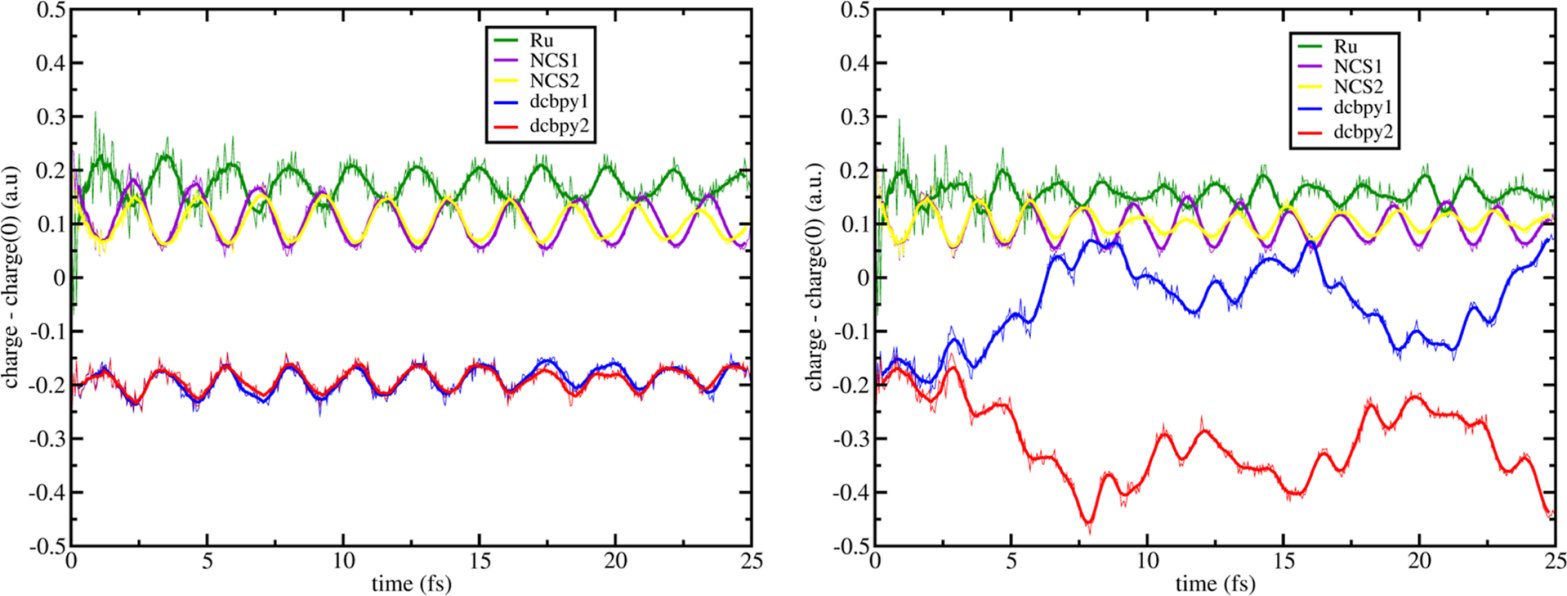
+0.025_**[a]**_ RT-TDB3LYP fragment charges difference dynamics (with respect to the S_0_ state) of states ^1^MLCT_*A*_ (left) and ^1^MLCT_*B*_ (right) for N3^4–^. Mulliken population analysis was performed every 50 as. Line smoothing was done through 10 steps window running averages. For fragment definitions, see legend and Figure 1.

The effects of **[a]** mode in both negative (resulting in the Ru–N(NCS) distance shortening) and positive (resulting in the Ru–N(NCS) distance elongation) *Q*_**[a]**_ values on ultrafast charge dynamics are reported in Figures 7 and 8, respectively. Negative displacement of **[a]** on ^1^MLCT_*A*_ (Figure 7, left panel) shows that the initial charge difference on NCS1 and NCS2 fragments indicates a less symmetric hole distribution with respect to the ground state minimum electronic dynamics (at *t* = 0, +0.19 a.u. and +0.25 a.u. for NCS1 and NCS2 fragments, respectively), although a similar electron localization on both dcbpy ligands is observed as in the reference (*Q*_**[i]**_ = 0) structure.

**Figure 9:**
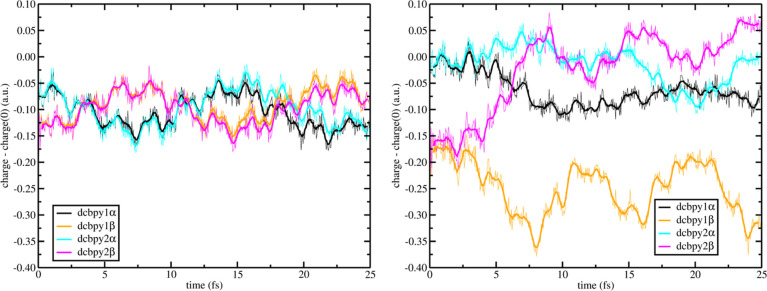
–0.025_**[a]**_ RT-TDB3LYP fragment charges difference dynamics (with respect to the S_0_ state) of states ^1^MLCT_*A*_ (left panel) and ^1^MLCT_*B*_ (right panel) for dcbpy ligands. α-aromatic rings are in trans to each other and β-aromatic rings are in trans to NCS^–^ ligands, see Figure 1 for fragment assignment. Mulliken population analysis were performed every 50 as. Line smoothing was done through 10 steps window running averages.

To better highlight the **[a]** mode effects, it is also analyzed the charge difference on each aromatic ring of both dcbpy ligands (we define with α and β the rings in cis, to both, and trans, at least for one, NCS ligands, respectively—see Figure 1). In Figure 9 left panel, is reported such analyses for the ^1^MLCT_*A*_. The electron is localized on both rings of both ligands, starting with a larger negative charge difference on the β portions (∼−0.19 a.u vs ∼−0.06 au). Interestingly, with a period of ∼3.5 fs, we observe an oscillating trend where the electron is moving independently from the β to α portion of each dcbpy ligands simultaneously, with no overall resulting neat exchange of charge between them, as inferred from Figure 7, left panel.

For the ^1^MLCT_*B*_ case (see Figure 7, right panel), the hole is still localized on the NCS and Ru fragments. The vibration induces the electron localization on both charge-accepting ligands (at *t* = 0: −0.24 a.u. for dcbpy1 and −0.22 a.u. for dcbpy2.), that after ∼2 fs starts to locate mostly on the dcbpy1 (blue line). Then, this difference increases, reaching the maximum at ∼8 fs, where dcbpy1 entirely localizes the electron (charge difference at *t* = 8 fs, of ∼−0.45 a.u.) and dcbpy2 (red line) loses the initial electron localization (charge difference at *t* = 8 fs, ∼ + 0.05 a.u.). This charge dynamics is quite different with respect to the symmetric ground state minimum previously described, since we observe a more rapid (less than ∼8 fs) ILET and no recombination after. As before, we repeat the dcbpy ring analysis that we report in Figure 9 right panel, for the ^1^MLCT_*B*_ case. In this case, the electron starts to be shared between both β portions of dcbpy ligands (orange and magenta lines), with no initial involvement of α rings. Around ∼ 2 fs, the α and β rings of each 1 and 2 charge-accepting ligands start to differentiate from each other, where now the dcbpy1 initiates to attract the electron more than the dcbpy2 (larger negative values of charge difference), as witnessed by the opposite trend of the dcbpy1β (orange line) and dcbpy2β (magenta line).

**Figure 10:**
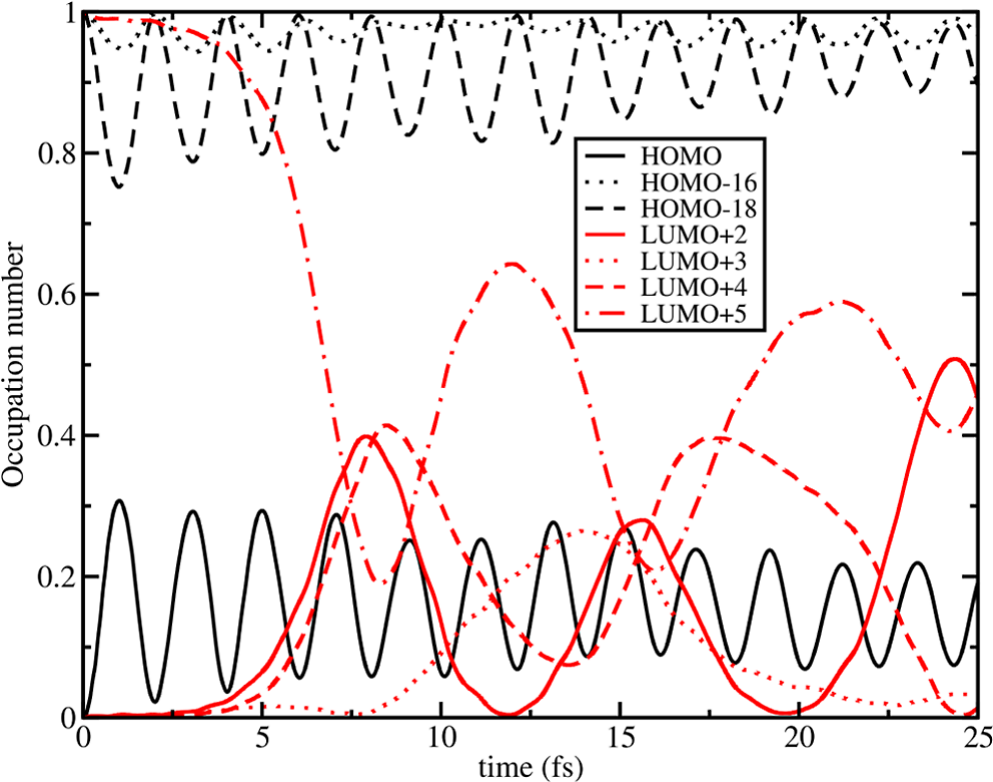
–0.025_**[a]**_ RT-TDB3LYP MO occupation number dynamics in N3^4–^ for state ^1^MLCT_*B*_ computed according to eq 6. Only alpha MO are reported. Individual frontier MO occupation number evolution is reported according to the legend in the graph, where in black and in red are represented the original occupied and virtual MOs for the ground state. Electronic Dynamics was prepared according to the MO swap procedure explained in the Computational Methods and Details section, where in this case the HOMO was emptied (full black line) and the LUMO+5 (dot-dashed red line) was populated instead at *t* = 0.

^1^MLCT_*A*_, under positive displacement of **[a]**, gives MO occupation number dynamics similar to the ground state minimum (Figure S18).

^1^MLCT_*B*_ of *Q*_**[a]**_ = +0.025 displaced structure shows a similar involvement of l+2 and l+4 MOs (starting also this time around ∼8 fs), with occupation dynamics very similar to the one seen in Figure 10 (see Figure 11 for comparison). Through LR-TDDFT analysis (see Table S7 and NTO maps in Figure S19), it has been found that the hole of ^1^MLCT_*B*_ spatially overlaps with the hole of lower energy states with respect to the previous case, in particular both S_20_ and S_21_ excitations are now involved. This observation is also true for the electron NTO of ^1^MLCT_*B*_ that spatially overlaps with electron NTO of S_20_ and S_21_ excitations, involving both dcbpy ligands (the character of these transitions is unchanged with respect to the ones observed for undistorted ground state equilibrium geometry, see Figures S2 and S19 for comparison).

Since RT-TDDFT simulations provide direct access to the MO occupation number dynamics, we can better interpret these results in terms of mixing and dynamically overlapping electronic states. In Figure 10 is shown the MO occupation number dynamics for ^1^MLCT_*B*_. We recall that this state is prepared by populating the l+5 orbital and depopulating the HOMO. Then, l+5 orbital starts to depopulate at ∼2 fs and consequently l+2 and l+4 begin to populate, reaching a maximum in the population around ∼8 fs which matches with the maximum charge difference observed for dcbpy fragments (see Figure 7, right panel). The presence of such l+2 and l+4 contributions is linked to the mixing of the original S_24_ state, with the largest ^1^MLCT_*B*_ character, with some additional and energetically close states where such orbitals might be involved, so we analyzed the LR-TDDFT data, noticing that such MOs are present in the excitation toward the immediately preceding electronic state S_23_ (see Table S6). Indeed, l+2 and l+4 (whose contribution is observed in S_23_ excitation) are localized on the α portions of dcbpy rings while l+5 (involved instead in the original ^1^MLCT_*B*_ excitation) is localized on the β portions of the rings. The nature of this transition has changed with respect to the undistorted equilibrium geometry, see Figures S3 and S17 for comparison. ^1^MLCT_*A*_ affected by negative displacement of **[a]** presents a very similar charge dynamics with respect to the ground state minimum equilibrium structure and its MO occupation number dynamics has no additional orbital pairs involvement as observed previously for ^1^MLCT_*B*_, instead (see Figure S16).

**Figure 11:**
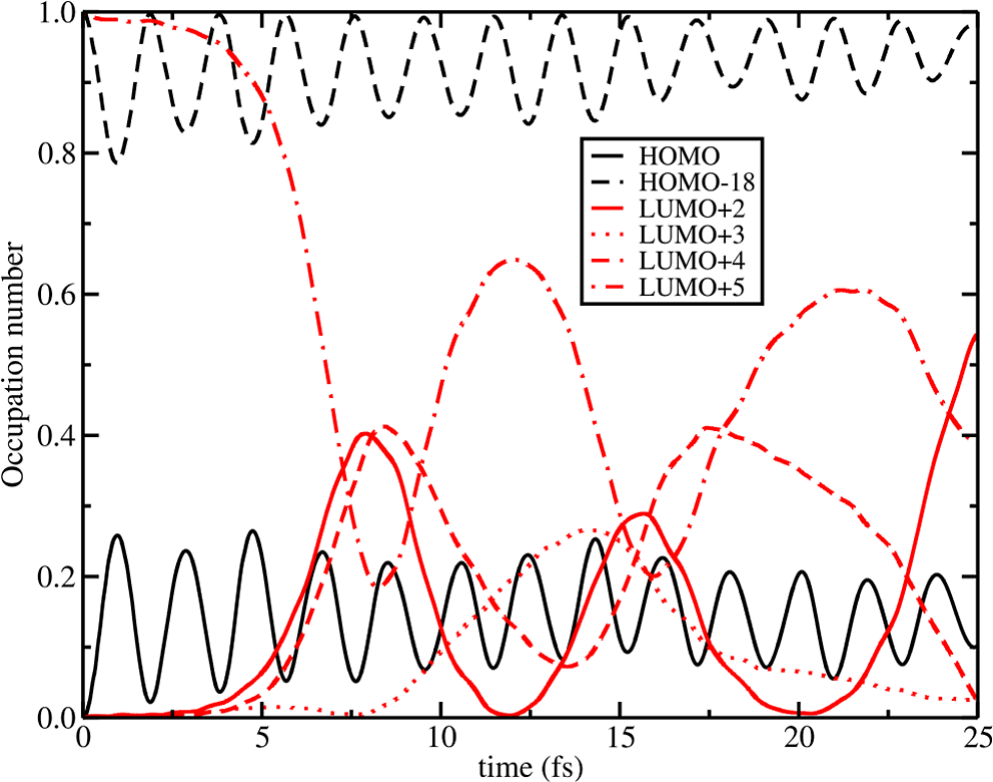
+0.025_**[a]**_ RT-TDB3LYP MO occupation number dynamics in N3^4–^ for state ^1^MLCT_*B*_ computed according to eq 6. Only alpha MO are reported. Individual frontier MO occupation number evolution is reported according to the legend in the graph, where in black and in red are represented the original occupied and virtual MOs for the ground state. Electronic dynamics was prepared according to the MO swap procedure explained in the Computational Methods and Details section, where in this case the HOMO was emptied (full black line) and the LUMO+5 (dot-dashed red line) was populated instead at *t* = 0.

**Figure 12:**
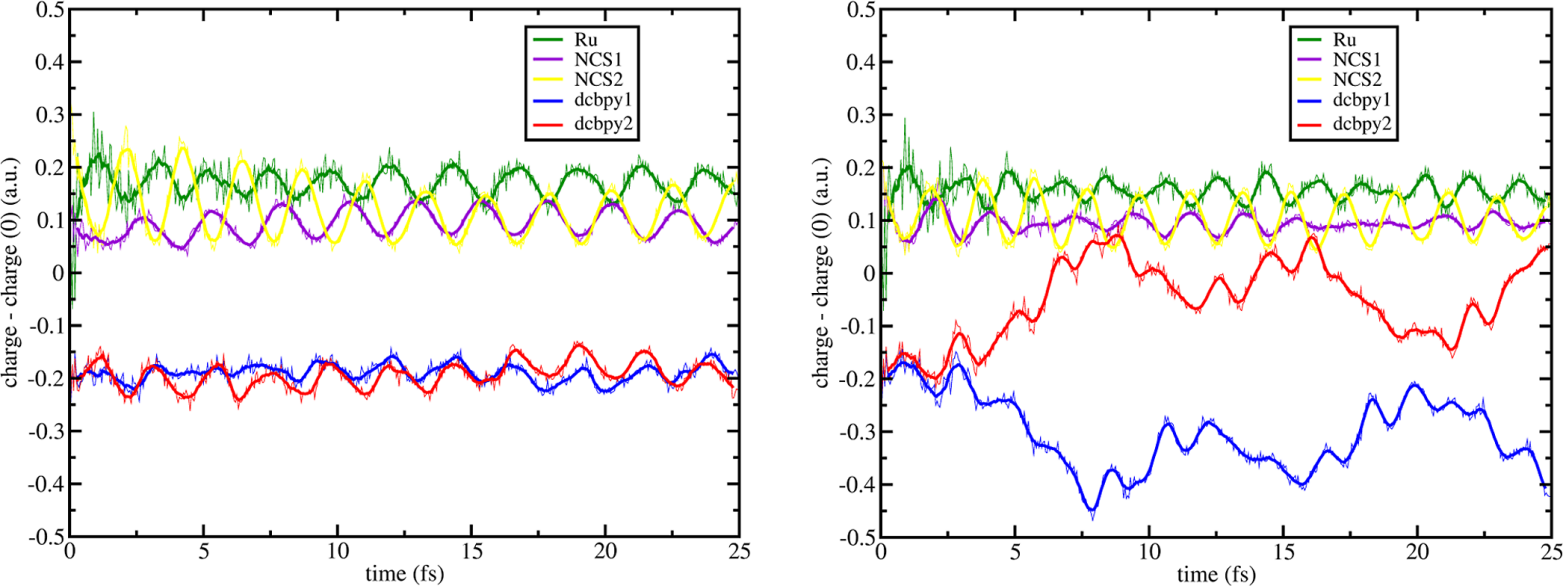
+0.025_**[b]**_ RT-TDB3LYP fragment charges difference dynamics (with respect to the S_0_ state) of states ^1^MLCT_*A*_ (left) and ^1^MLCT_*B*_ (right) for N3^4–^. Mulliken population analysis was performed every 50 as. Line smoothing was done through a 10-step window running averages. For fragment definitions, see legend and Figure 1.

Vibrational mode **[b]** (see Figure 2 for its definition), gives similar effects on ^1^MLCT_*A*_ and ^1^MLCT_*B*_ charge dynamics, as seen for vibration **[a]**. The hole is located on the metal center and NCS^–^ ligands and the electron is located on both dcbpy fragments (Figure 12). On ^1^MLCT_*A*_ (see Figure 12, left panel), it was noted that charge dynamics of both NCS and dcbpy fragments are more asymmetrical compared to **[a]**, although subsequently at ∼10 fs hole and electron localization became more symmetric. Regarding the higher energy MLCT state, we observe that both **[a]** and **[b]** modes (that involve Ru-NCS distance) speed up the ILET process with no recombination after on for the ^1^MLCT_*B*_ excitation with respect to the equilibrium structure (see Figure 12, right panel).

Also, for such vibration, the MO occupation number dynamics for ^1^MLCT_*A*_ and ^1^MLCT_*B*_ was investigated (see Figures S20 and S21). **[b]** mode, involving as the **[a]** mode the Ru–N(NCS) distance, gives similar effects with respect to the previous distortions on time-dependent MO occupation dynamics of ^1^MLCT_*A*_, also similar to the ground state minimum, and ^1^MLCT_*B*_, showing for this last case the involvement of l+2 and l+4 starting at ∼8 fs (see Figures 10, 11, S16 and S18 and S20 and S21). LR-TDDFT analysis highlights (Table S8), as observed for **[a]** positive displacement, that the hole and electron of ^1^MLCT_*B*_ combines with S_20_ and S_21_ electronic states. As for the **[a]** positive displaced geometry, the character of these transitions is unchanged with respect to the ones observed for undistorted ground state equilibrium geometry, see Figures S2 and S19 for comparison.

**Figure 13:**
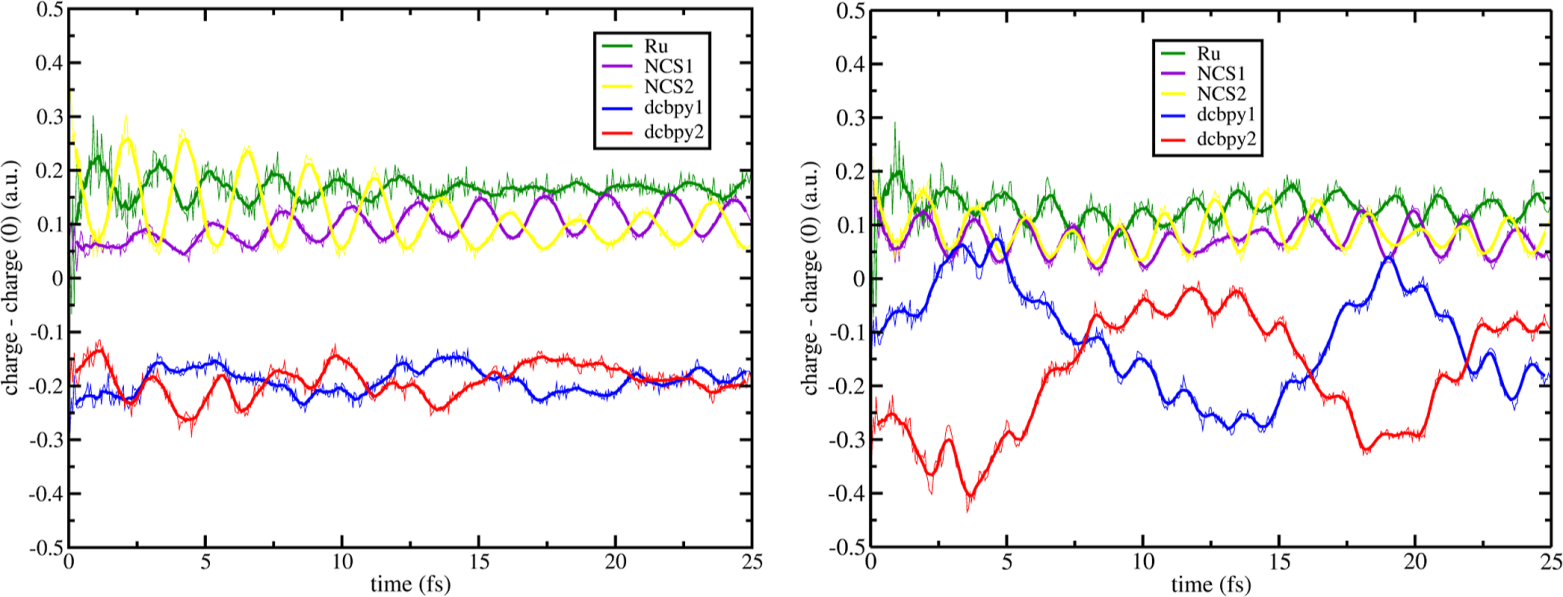
+0.18_**[c]**_ RT-TDB3LYP fragment charge difference dynamics (with respect to the S_0_ state) of ^1^MLCT_*A*_, left, and ^1^MLCT_*B*_, right, for N3^4–^. Mulliken population analysis was performed every 50 as. Line smoothing was done through 10 steps window running averages. For fragment definitions, see legend and Figure 1.

Unlike **[a]** and **[b]** vibrational modes, which involve Ru–N(NCS) distances, vibration **[c]** affects the dcbpy–dcbpy distance (see Figure 2, third panel). The effects of **[c]** + 0.18 displacement on ultrafast charge dynamics are reported in Figure 13. As previously shown, vibration **[c]** affects the MLCT character of the adiabatic states, and now states S_18_ and S_23_ have the largest correspondence to ^1^MLCT_*A*_ and ^1^MLCT_*B*_, respectively (see Figure S12). This behavior does not change the orbital contributions to the examined MLCT excitations, and we performed the same orbital swaps (as for the previous cases) for RT-TDDFT analysis: S_18_, h–1 → l+2 and S_23_, h → l+5.

+0.18_**[c]**_ displacement on ^1^MLCT_*A*_ ED also gives a different location of the hole and electron with respect to the ground state minimum. At *t* = 0, the hole is mostly located on NCS2 fragment (∼+0.36 a.u., yellow line Figure 13, left panel), and the resulting hole dynamics (NCS1, NCS2, and Ru) is initially mostly localized on this fragment, although during the ED, this effect is mitigated. Electron localization is initially still centered on both dcbpy ligands (at *t* = 0, dcbpy1: ∼−0.29 a.u. and dcbpy2: ∼−0.20 a.u.), as observed for the ground state minimum. Charge dynamics of dcbpy1 (blue line Figure 13, left panel) and dcbpy2 (red line Figure 13, left panel) shows that the electron little oscillates between the dcbpy fragments with no overall resulting neat interligand electron migration. This behavior is also confirmed by the individual α and β aromatic rings charge difference dynamics (see Figure S22). ^1^MLCT_*A*_ shows indeed: (i) for the **[a]**-affected ED a regular behavior as for the nondistorted minimum geometry; (ii) for the **[b]**-affected ED, already a slightly different charge dynamics, since one NCS ligand is closer than the other one, given the nature of the asymmetric vibration; (iii) for the **[c]**-affected ED, a more evident asymmetric nature of the hole localization, that is mostly located on Ru and NCS2, with almost null contribution on NCS1.

Time-dependent fragment charge difference of ^1^MLCT_*B*_ ED, see Figure 13 right panel, shows that the hole is mostly shared for the first ∼10 fs between both NCS fragments. Electron is mostly located, at *t* = 0, on the dcbpy2 fragment (∼−0.33 a.u.). At ∼8 fs, we assist to an ILET process that occurs at earlier times than the ones observed on the undistorted ground state minimum (∼15 fs, see ref (24)). Interestingly, we observe in the ED for the **[c]**-affected ^1^MLCT_*B*_ a periodic ILET, with a period of ∼8 fs, during which the electron jumps back and forth between the dcbpy ligands. We remind here that for both **[a]** and **[b]**-affected ^1^MLCT_*B*_ EDs, an ILET within 2.5 fs with no further oscillation of the electron among the dcbpy ligands was present, instead. We also analyzed the charge difference dynamics on the dcbpy rings (see Figure S23) for ^1^MLCT_*B*_. Unlike **[a]**-affected ^1^MLCT_*B*_ dcbpy ring analysis, **[c]**-affected ED sees a more significant involvement of both α and β aromatic rings of the dcbpy ligands. At *t* = 0 the α-rings (dcbpy1α, ∼0.00 a.u. and dcbpy2α, ∼−0.01 a.u.) show a less negative charge difference with respect to the β-rings (dcbpy1β, ∼−0.13 a.u. and dcbpy2β, ∼−0.32 a.u.). Along the charge dynamics, for the first ∼5 fs, the electron is mostly located on the dcbpy2β ring. As soon as the electron starts to oscillate on both dcbpy ligands (and so for the remaining dynamics), all α- and β-rings are involved in the ILET processes. MO occupation number dynamics for **[c]**-affected ^1^MLCT_*A*_ is similar to the ground state minimum and also to **[a]**- and **[b]**-affected ^1^MLCT_*A*_ electronic dynamics (see Figure S24). On the other hand, MO occupation number time evolution for **[c]**-affected ^1^MLCT_*B*_ presents a more involved dynamics (see Figure S25), with respect to the previous cases. Indeed, while l+5 begins to depopulate, consequently, at ∼5 fs l+2 has the maximum value in population (such a time matches with the first, and larger charge difference maximum observed for dcbpy fragment charge dynamics, see Figure 13, right panel) and for the remaining dynamics follows the same pattern. Through NTO and RT-TDDFT charge dynamics, we noted that MO l+2 involves both α and β rings in dcbpy ligands (see Figure S26) confirming the charge dynamics evolution in Figure S23. By the analysis of LR-TDDFT data (see Table S9), we observed that MO l+2 is present in the excitation toward the S_20_ and S_22_ states, indicating the mixing of S_23_ with them in contributing to the ^1^MLCT_*B*_ as consequence of **[c]** vibrational mode (the nature of S_20_ transition has changed with respect to the undistorted equilibrium geometry, see Figures S2 and S27 for comparison).

## 4 Conclusions

In this work, an innovative theoretical-computational protocol combining LR-TDDFT formalism, to characterize excitation energies and spacing among electronic levels (the electronic layouts), and real-time TDDFT, to understand the ultrafast (femtosecond) charge dynamics on the molecular scale, is presented and validated. Such a development is applied for the detailed study of photoinduced MLCT states (belonging to the 372 nm band) of a Ru(II) complex and their interplay with peculiar nuclear vibrations. *Via* real-time time-dependent density functional theory electronic dynamics, a molecular picture of the ultrafast (femto-second) hole–electron dynamics in time has been unraveled to highlight the role of such nuclear motions on the CT dynamics and ILET. For this aim, in this work we extensively studied three important vibrational modes that can affect either the Ru-(NCS) charge-donor segment or the dcbpy charge-acceptor portion on the N3^4–^ molecule. As the main result, we observed that such MLCT states are strongly influenced by structural distortions derived by all chosen vibrations. Regarding the ^1^MLCT_*A*_, we observe a regular behavior as for the nondistorted minimum geometry for **[a]**-affected dynamics. Then, looking at **[b]**-affected, the two NCS show already slightly different charge dynamics, since one NCS ligand is closer than the other one, given the nature of the asymmetric vibration. Regarding the **[c]**-affected, the asymmetric nature of the hole localization is more evident, which is mostly located on Ru and NCS2, with almost a null contribution on NCS1. A corresponding reorganization of the hole in [b] and [c] case studies, leading for both to the hole randomization and a similar phase, is observed for both at the end. Concerning the ^1^MLCT_*B*_, a faster interligand electron transfer mechanism for the **[c]**-affected ED, or a very asymmetric electron delocalization for the **[a]**- and **[b]**-affected EDs is observed. Additionally, a large mixing of such MLCT states with the energetically close electronic states was observed for all analyzed vibrations thanks to the combinations of LR-TDDFT results and the molecular pictures of CT dynamics provided by RT-TDDFT MO occupation time evolution. For both of the electronic states with the larger ^1^MLCT_*A*_ and ^1^MLCT_*B*_ characters, the mixing with several preceding states is observed that influences also the electron localization in time (randomizing its distribution on both α and β rings portions) for all vibrations. Additionally, the motion affecting the electron-accepting regions shows an energetic inversion of the adiabatic electronic state with a larger MLCT character along this **[c]** vibration. We believe that the presented protocol and results will motivate other groups to utilize RT-TDDFT simulations for testing and designing better performing dye sensitizers to exploit the role of nuclear motions in the ultrafast charge dynamics that rule the charge injection into substrates. As future directions, explicit nuclear wave packet dynamics could be exploited along with Ehrenfest molecular dynamics to further investigate such phenomena on a longer time-scale. Such results will improve the molecular interpretation of multidimensional vibro-electronic spectroscopic techniques, used to characterize ultrafast coherent and noncoherent dynamics of complex systems, and will help the enhancement of dyes performances.

## Supporting Information Available

Analysis of N3^4–^ structural parameters from MD simulations at room temperature; MO and NTO representations; Walsh diagrams, transition electric dipole strength, and energy derivatives along the case study electronic states under vibrational distortions; LR-TDB3LYP vertical excitation energies on N3^4–^ vibrationally distorted structures; and RT-TDB3LYP fragment charge difference for dcbpy ligands and MO occupation number dynamics for ^1^MLCT_*A*_ and ^1^MLCT_*B*_ on vibrationally distorted structures (PDF)
