# Supplementary material for: Watching the Interplay between Photoinduced Ultrafast Charge Dynamics and Nuclear Vibrations
Source: J Chem Theory Comput. 2023 Nov 22;19(23):8751–66. doi: 10.1021/acs.jctc.3c00855 (PMC10720350; doi:10.1021/acs.jctc.3c00855)
Supplement: Supplementary file 1 — ct3c00855_si_001.pdf [file ct3c00855_si_001.pdf]

Electronic Supplementary Information: Watching the Interplay between Photoinduced Ultrafast Charge Dynamics and Nuclear Vibrations

Edoardo Buttarazzi,[†,‡] Fulvio Perrella,[†] Nadia Rega,[‡,†,¶] and Alessio Petrone[*,‡,†,¶]

†*Scuola Superiore Meridionale, Largo San Marcellino 10, I-80138, Napoli, Italy*

‡*Department of Chemical Sciences, University of Napoli Federico II, Complesso Universitario di Monte S. Angelo, via Cintia 21, I-80126, Napoli, Italy*

¶*Istituto Nazionale Di Fisica Nucleare, sezione di Napoli, Complesso Universitario di Monte S. Angelo ed. 6, via Cintia, I-80126, Napoli, Italy*

E-mail: alessio.petrone@unina.it

1 Investigation of geometrical parameters of N3^{4-} through MD trajectory

1.1 Vibration [a] and [b] displacement range choice

Table S1: Vibration [**a**] AIMD study of the variation of the distance length Ru-N(NCS) through several vibrational displacements. $\sigma = \pm 0.055$ Å; maximum distance length: 2.269 Å; minimum distance length: 1.922 Å; average value: 2.064 Å. ±0.025 displacement range was chosen.

Vibrational displacement	Distance length Ru-N (Å)
-0.025	2.068
-0.020	2.068
-0.015	2.067
-0.010	2.067
-0.005	2.067
0.000	2.066
+0.005	2.064
+0.010	2.062
+0.015	2.060
+0.020	2.058
+0.025	2.056

Table S2: Vibration [**b**] AIMD study of the variation of the distance length Ru-N(NCS) through several vibrational displacements. $\sigma = \pm 0.055$ Å; maximum length value: 2.269 Å; minimum length value: 1.922 Å; average value: 2.064 Å. ±0.025 displacement range was chosen.

Vibrational displacement	Distance length Ru-N (Å)
-0.025	2.077
-0.020	2.075
-0.015	2.073
-0.010	2.071
-0.005	2.068
0.000	2.066
+0.005	2.064
+0.010	2.062
+0.015	2.060
+0.020	2.058
+0.025	2.056

1.2 Vibration [c] displacement range choice

Table S3: Vibration [**c**] AIMD study of the variation of the distance length Ru-N(NCS) through several vibrational displacements. $\sigma = \pm 0.055$ Å; maximum length value: 2.269 Å; minimum length value: 1.922 Å; average value: 2.064 Å. ±0.4 displacement range was chosen.

Vibrational displacement	Distance length Ru-N (Å)
-1.0	2.190
-0.8	2.164
-0.6	2.138
**-0.4**	**2.113**
**-0.2**	**2.089**
**0.0**	**2.066**
**+0.2**	**2.089**
**+0.4**	**2.113**
+0.6	2.138
+0.8	2.164
+1.0	2.190

Table S4: Vibration [**c**] AIMD study of the variation of the distance length N(NCS)-N(dcbpy) through several vibrational displacements. $\sigma = \pm 0.101$ Å; maximum length value: 2.648 Å; minimum length value: 3.279 Å; average value: 2.951 Å. ±0.4 displacement range was chosen.

Vibrational displacement	Distance length N-N (Å)
-1.0	2.702
-0.8	2.745
-0.6	2.788
**-0.4**	**2.831**
**-0.2**	**2.875**
**0.0**	**2.918**
**+0.2**	**2.875**
**+0.4**	**2.831**
+0.6	2.788
+0.8	2.745
+1.0	2.702

Table S5: Vibration [**c**] AIMD study of the variation of the N-Ru-N angle (dcbpy-Ru-NCS cis-positioned) through several vibrational displacements. $\sigma = \pm 3.47\,^{\circ}$; maximum angle value: 102.55 °; minimum angle value: 81.28 °; average value: 90.91 °. ±0.4 displacement range was chosen.

Vibrational displacement	Angle N-Ru-N (°)
-1.0	102.30
-0.8	99.67
-0.6	97.05
**-0.4**	**94.45**
**-0.2**	**91.86**
**0.0**	**89.30**
**+0.2**	**91.86**
**+0.4**	**94.45**
+0.6	97.05
+0.8	99.67
+1.0	102.30

2 Electronic layout on ground state minimum energy structure

2.1 MO analysis of S_{19} and S_{24}

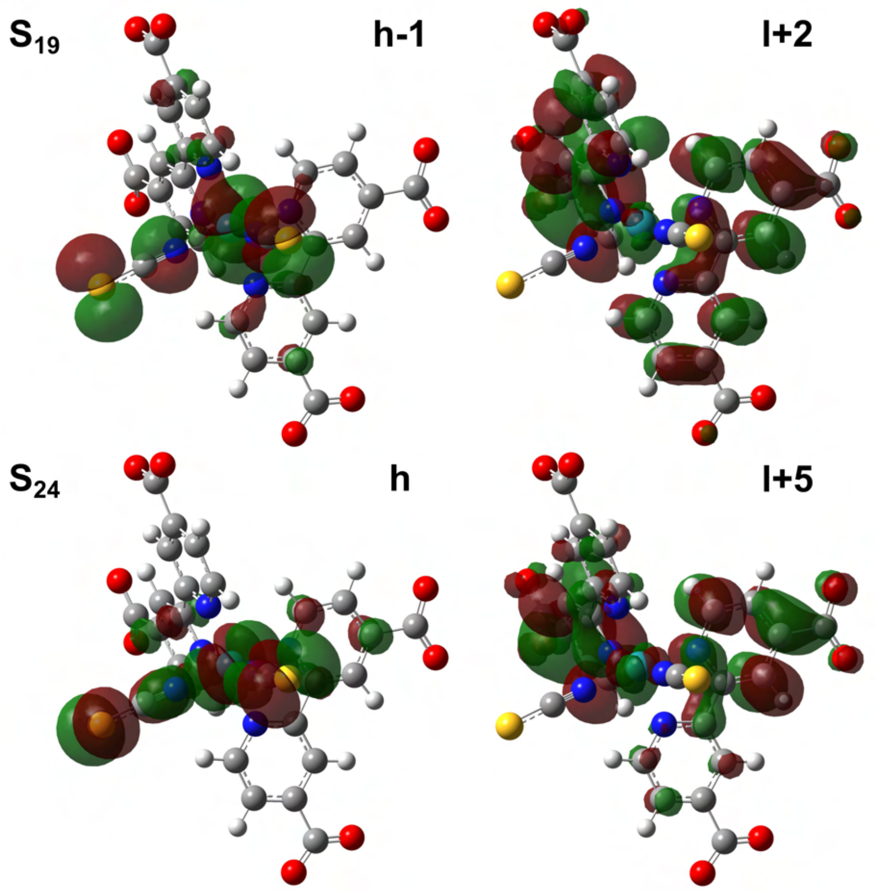

Figure S1: MO pairs with larger contributions in the electronic transitions towards the S_{19} and S_{24} adiabatic states. Isovalue: 0.02.

2.2 NTO analysis of the several electronic states, from S_{17} to S_{27}

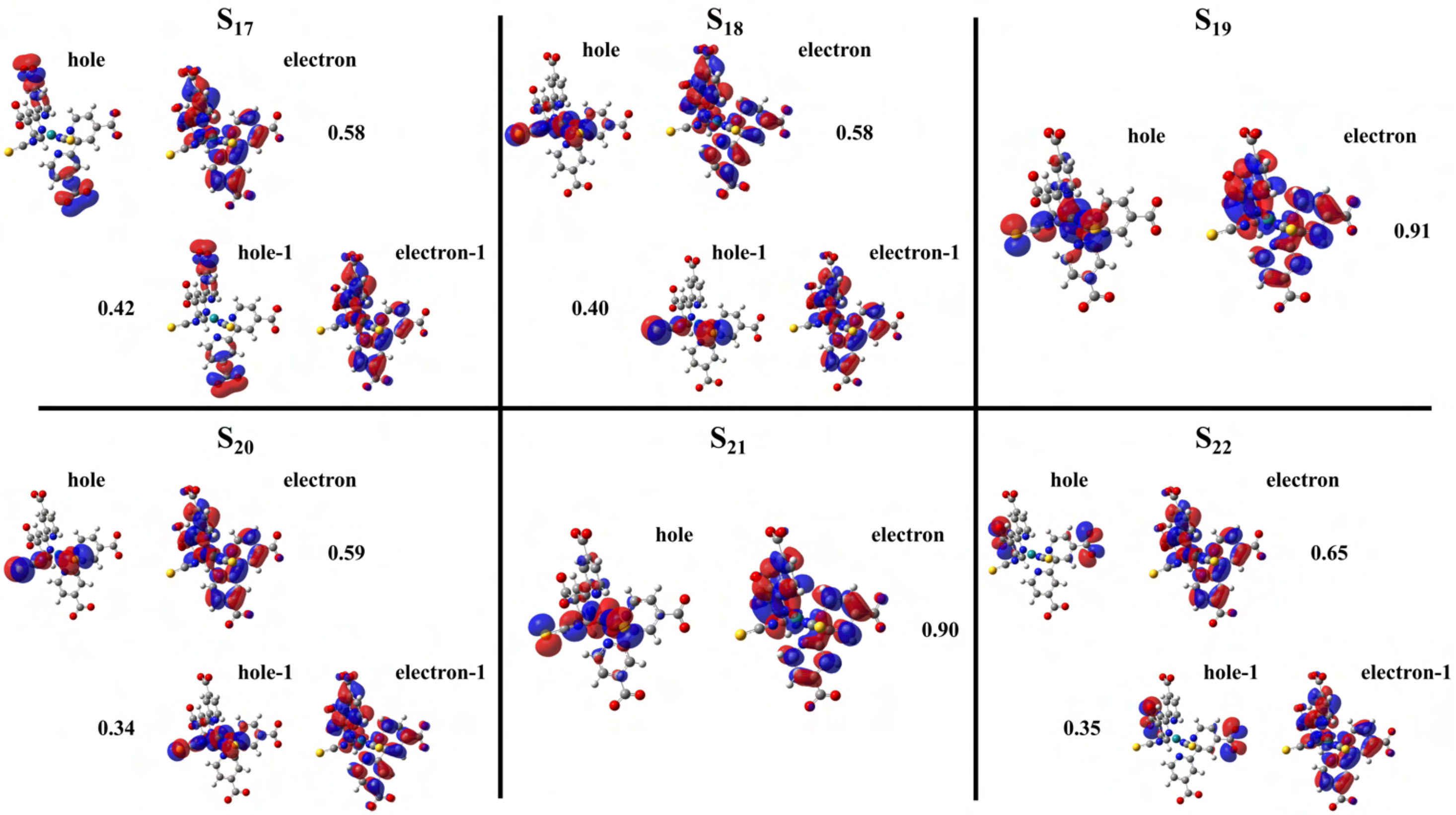

Figure S2: Hole and electron NTOs for the electronic transitions towards the analyzed adiabatic excited electronic states (from S_{17} to S_{22}) for the equilibrium geometry. Occupation numbers are reported in the figure. Isovalue: 0.02.

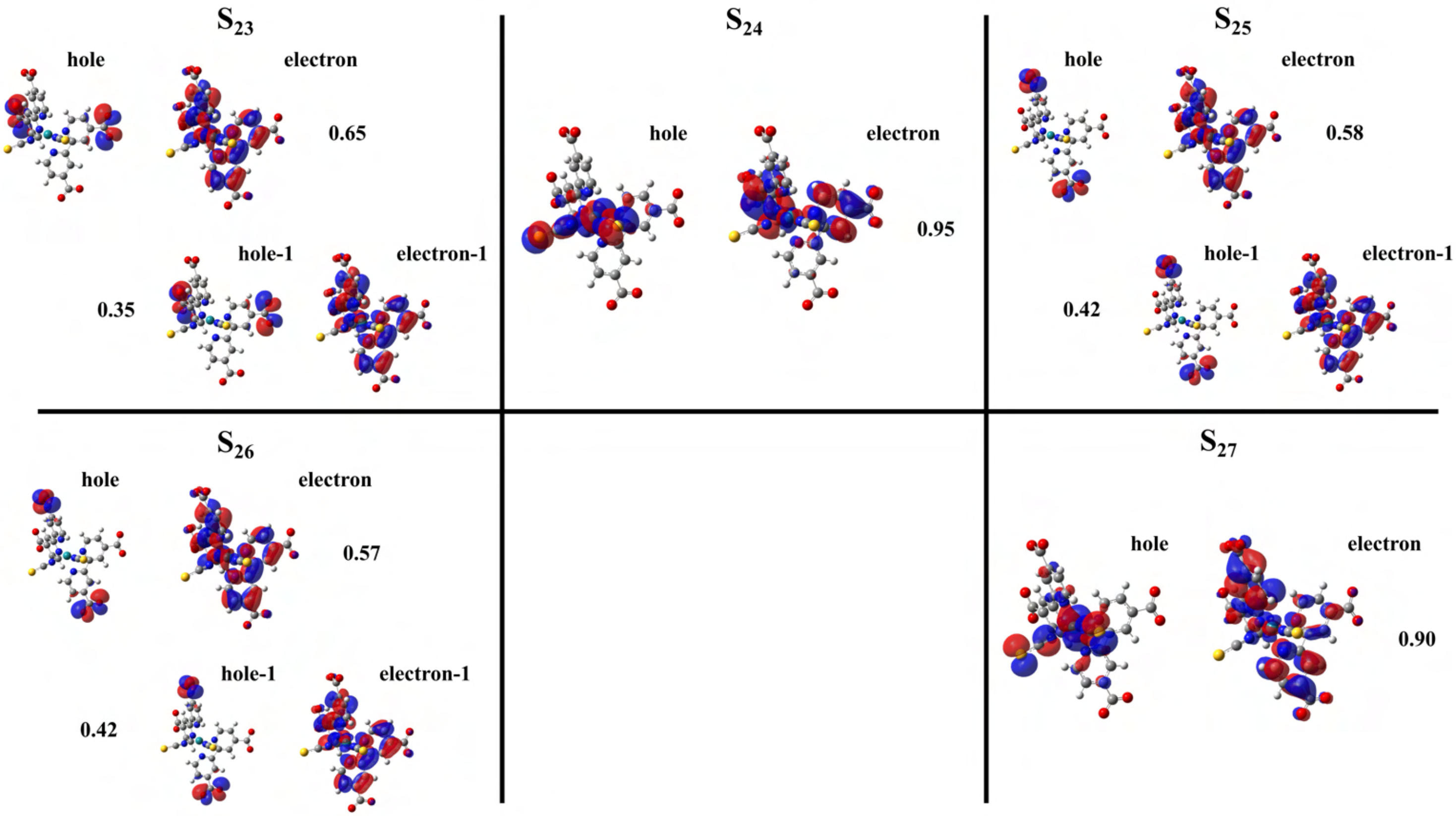

Figure S3: Hole and electron NTOs for the electronic transitions towards the analyzed adiabatic excited electronic states (from S_{23} to S_{27}) for the equilibrium geometry. Occupation numbers are reported in the figure. Isovalue: 0.02.

3 Vibrational effects on high energy electronic MLCT states and electronic manifold

3.1 NTO analysis vibration [a]

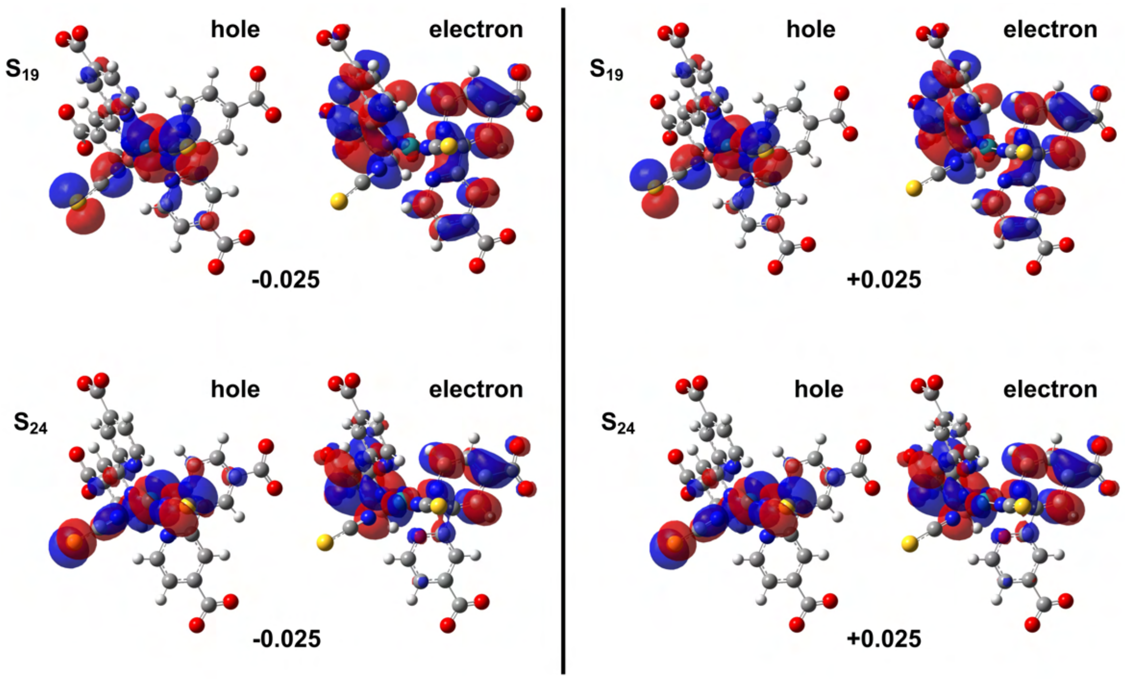

Figure S4: Hole and electron NTOs for the electronic transitions towards the adiabatic S_{19} and S_{24} electronic states under ±0.025 displacement values distorted by [**a**]. Isovalue: 0.02.

3.2 Walsh diagrams vibration [a]

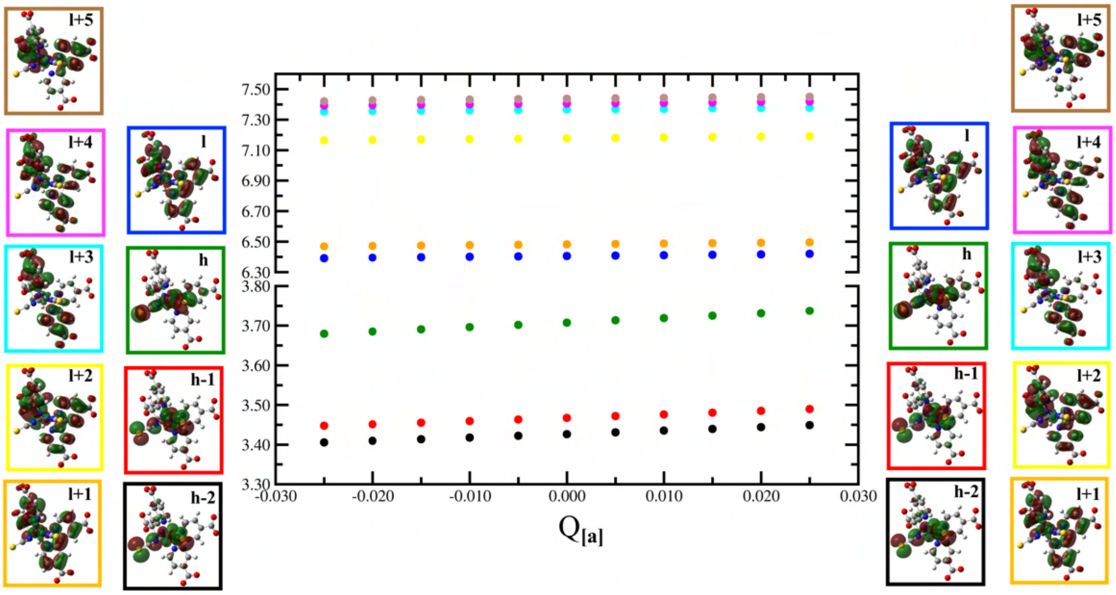

Figure S5: MO Walsh diagrams under [**a**]-vibrational distortion ($Q_{[a]} = \pm 0.025$ range, 0.005 step). HOMO-2, black dots; HOMO-1, red dots; HOMO, green dots; LUMO, blue dots; LUMO+1, orange dots; LUMO+2, yellow dots; LUMO+3, turquoise dots; LUMO+4, magenta dots and LUMO+5, brown dots. Energy is reported in eV. MO isovalue: 0.02.

3.3 Transition electric dipole strength of the several electronic states influenced by [a]

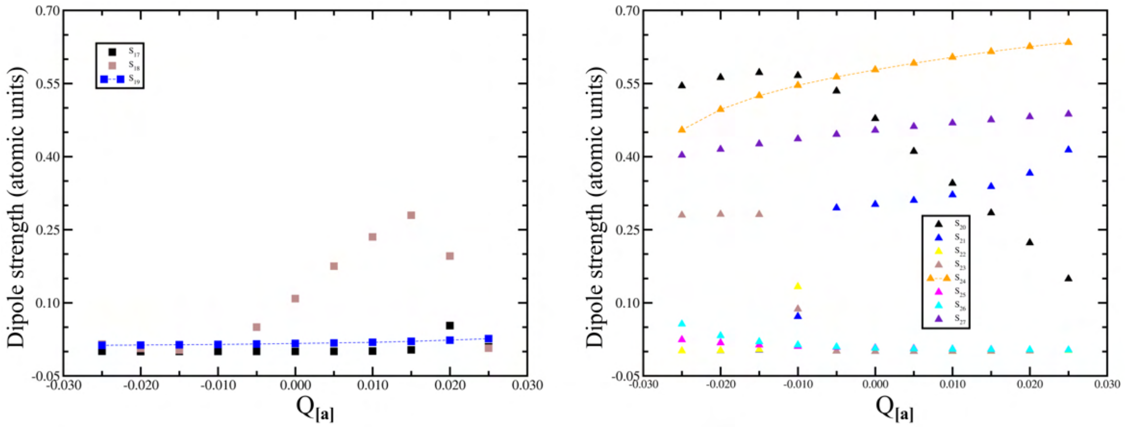

Figure S6: LR-TDDFT ground state to excited states (from S_{17} to S_{27}) transition electric dipole strengths (expressed as magnitude squared) under [**a**]-distortion. Left, from S_{17} to S_{19} adiabatic excited states; right, from S_{20} to S_{27} adiabatic excited states.

3.4 Energy derivatives of S_{19} and S_{24} states under [a] distortion

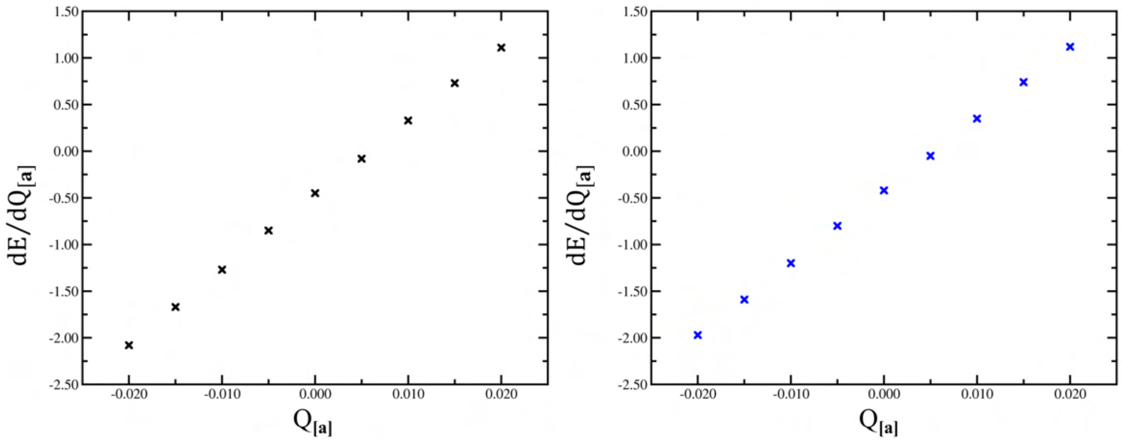

Figure S7: Energy derivatives of S_{19} (left panel) and S_{24} (right panel) adiabatic electronic states under [**a**] vibrational distortion. At $Q_{[a]} = -0.025$ displacement, $dE/dQ = -2.28\,\text{eV}$ for S_{19} state and $dE/dQ = -2.16\,\text{eV}$ for S_{24} (data not displayed on graph but obtained *via* backward and forward difference method, respectively). At $Q_{[a]} = +0.025$ displacement, $dE/dQ = +1.30\,\text{eV}$ and $dE/dQ = 1.32\,\text{eV}$ for S_{19} and S_{24}, respectively (data not displayed on graph but obtained through backward and forward difference method, respectively). Values are expressed as eV. Derivatives are obtained through numerically differentiating the energy values *via* the central difference method.

3.5 NTO analysis vibration [b]

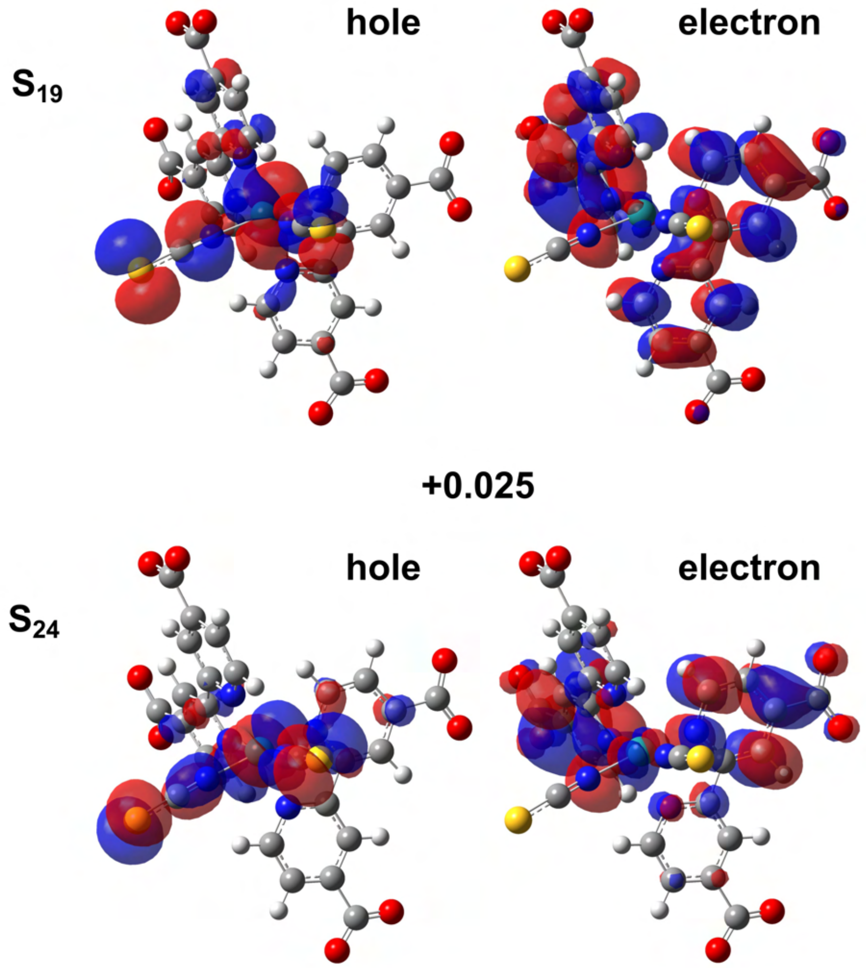

Figure S8: Hole and electron NTOs for the electronic transitions towards the adiabatic S_{19} and S_{24} under +0.025 displacement value distorted by [**b**]. Isovalue: 0.02.

3.6 Walsh diagrams vibration [b]

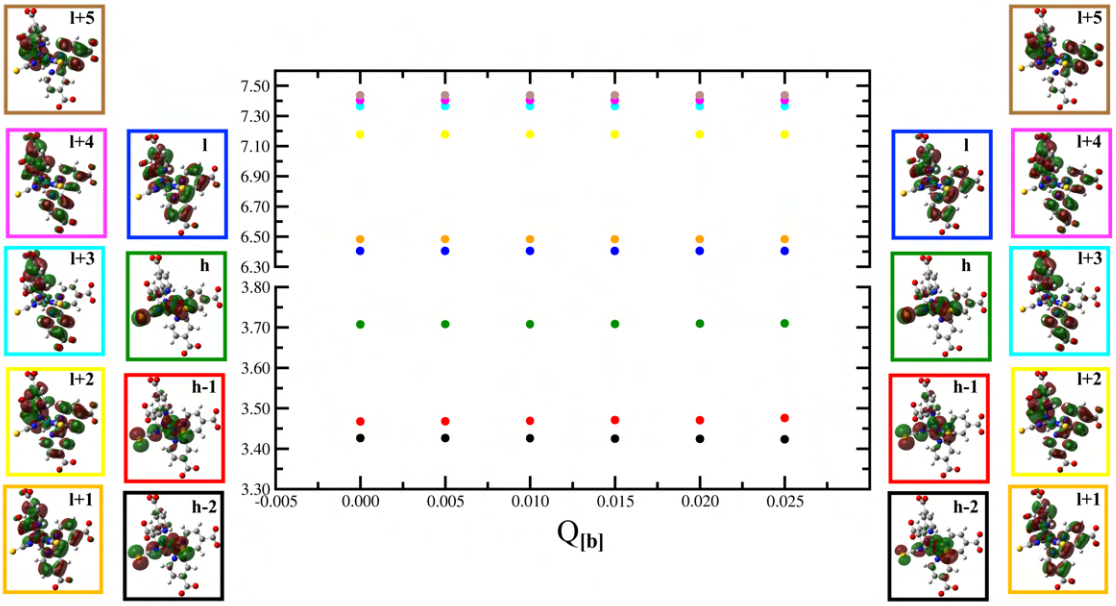

Figure S9: MO Walsh diagrams under [**b**]-vibrational distortion ($Q_{[b]}$ from 0 to 0.025 range, 0.005 step). HOMO-2, black dots; HOMO-1, red dots; HOMO, green dots; LUMO, blue dots; LUMO+1, orange dots; LUMO+2, yellow dots; LUMO+3, turquoise dots; LUMO+4, magenta dots and LUMO+5, brown dots. Energy is reported as eV. MO isovalue: 0.02.

3.7 Transition electric dipole strength of the several electronic states influenced by [b]

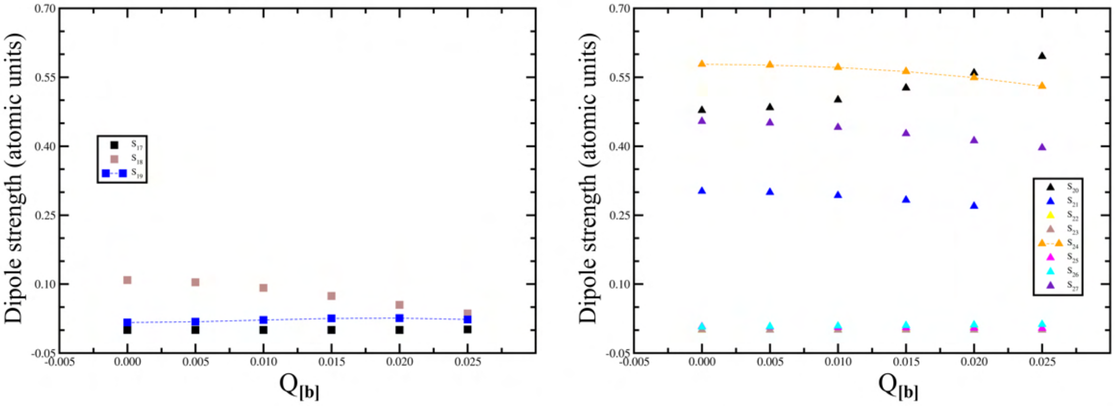

Figure S10: LR-TDDFT ground state to excited states (from S_{17} to S_{27}) transition electric dipole strengths (expressed as magnitude squared) under [**b**]-distortion. Left, from S_{17} to S_{19} adiabatic excited states; right, from S_{20} to S_{27} adiabatic excited states.

3.8 Energy derivatives of S_{19} and S_{24} states under [b] distortion

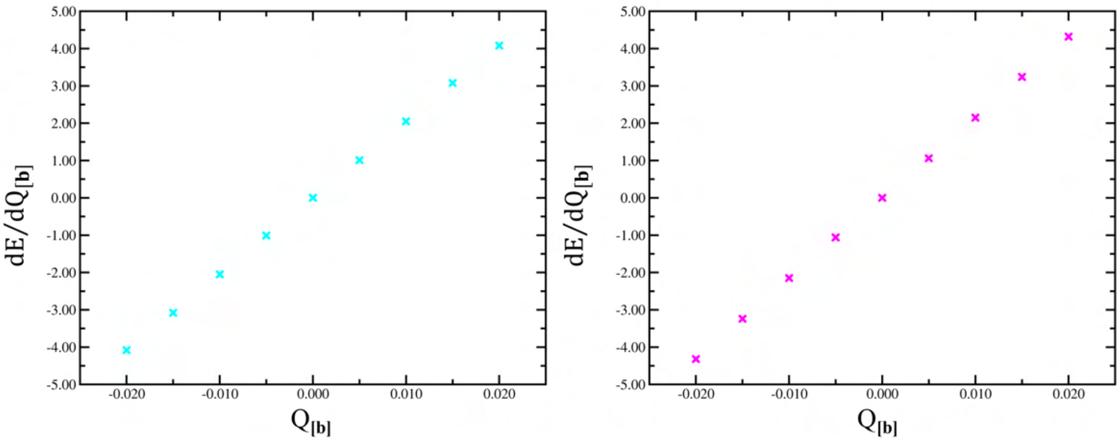

Figure S11: Energy derivatives of S_{19} (left panel) and S_{24} (right panel) adiabatic electronic states under [**b**] vibrational distortion. At $Q_{[b]} = +0.025$ displacement, $dE/dQ = +4.56\,\text{eV}$ for S_{19} state and $dE/dQ = +4.86\,\text{eV}$ for S_{24} (data not displayed on graph but obtained *via* backward and forward difference method, respectively). Values are expressed as eV. Derivatives are obtained through numerically differentiating the energy values *via* the central difference method.

3.9 NTO analysis vibration [c]

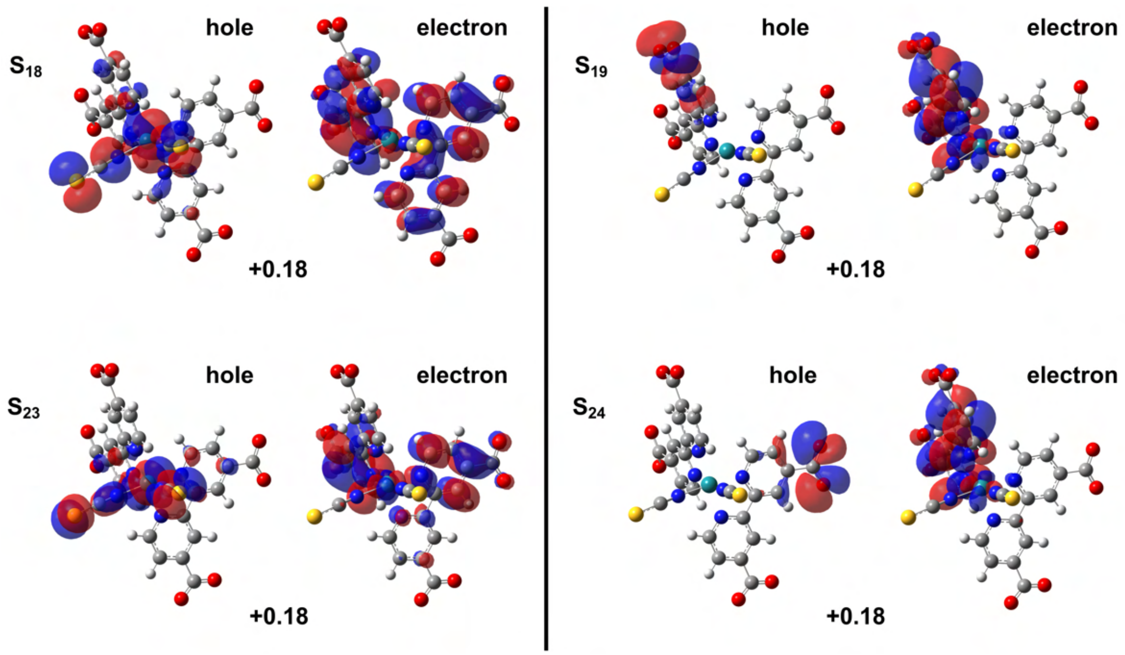

Figure S12: Hole and electron NTOs for the electronic transitions towards the adiabatic S_{19}, S_{24}, S_{18} and S_{23} electronic states under +0.18 displacement value distorted by [**c**]. Isovalue: 0.02.

3.10 Walsh diagrams vibration [c]

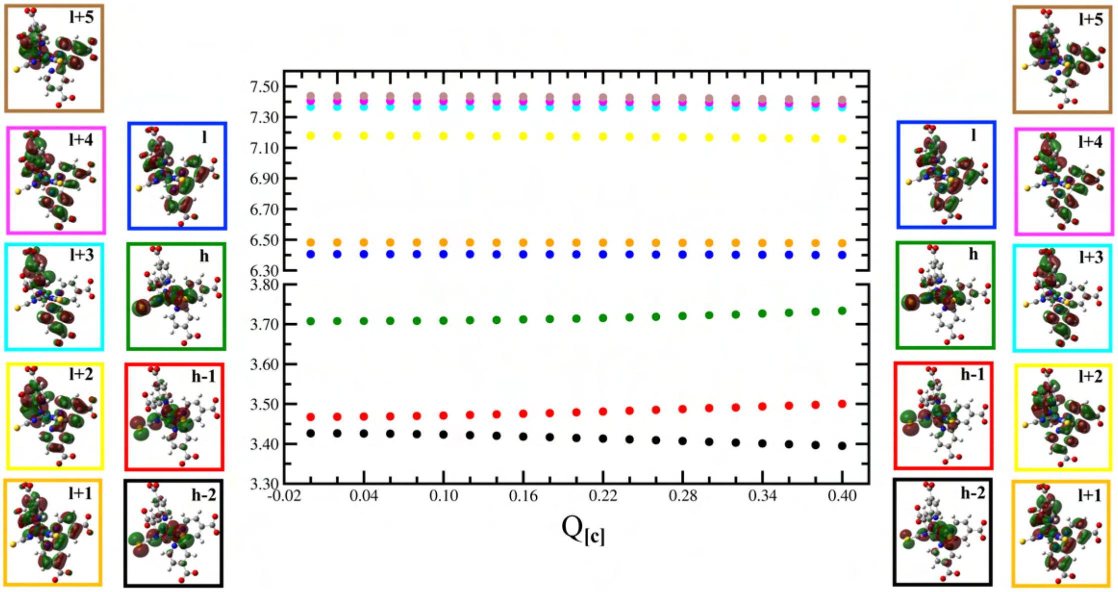

Figure S13: MO Walsh diagrams under [**c**]-vibrational distortion ($Q_{[c]}$ from 0 to +0.40 range, 0.02 step). HOMO-2, black dots; HOMO-1, red dots; HOMO, green dots; LUMO, blue dots; LUMO+1, orange dots; LUMO+2, yellow dots; LUMO+3, turquoise dots; LUMO+4, magenta dots and LUMO+5, brown dots. Energy is reported as eV. MO isovalue: 0.02.

3.11 Transition electric dipole strength of the several electronic states influenced by [c]

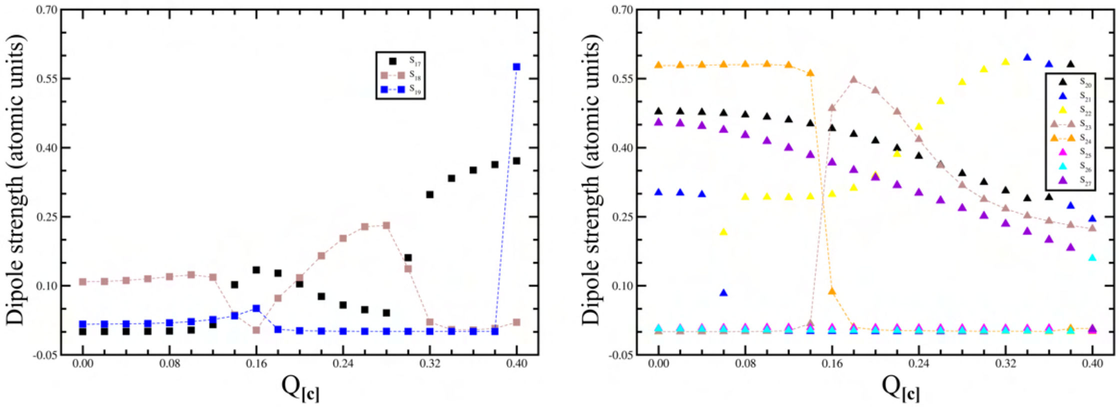

Figure S14: LR-TDDFT ground state to excited states (from S_{17} to S_{27}) transition electric dipole strengths (expressed as S^2) under [**c**]-distortion. Left, from S_{17} to S_{19} adiabatic excited states; right, from S_{20} to S_{27} adiabatic excited states.

3.12 Energy derivatives of S_{18} and S_{23} states under [c] distortion

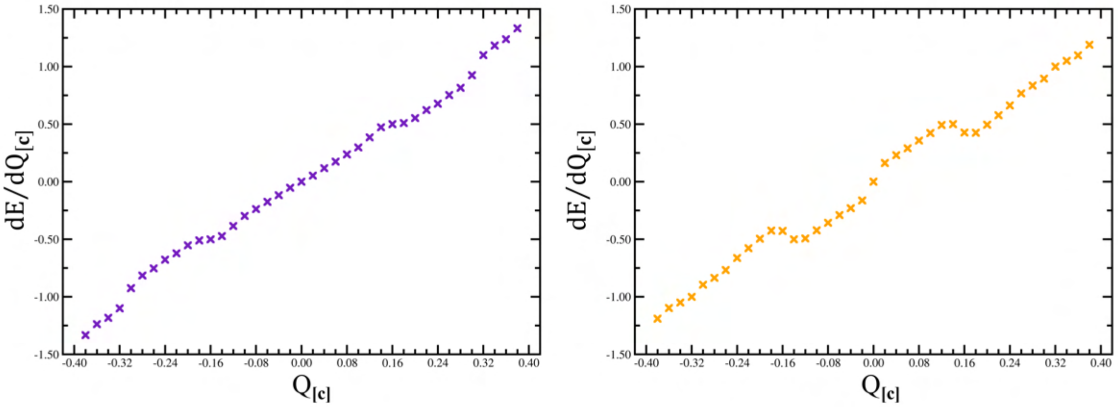

Figure S15: Energy derivatives of S_{18} (left panel) and S_{23} (right panel) adiabatic electronic states under [**c**] vibrational distortion. At $Q_{[c]} = +0.18$ displacement, $dE/dQ = +0.50\,\text{eV}$ for S_{18} state and $dE/dQ = +0.43\,\text{eV}$ for S_{23} (data not displayed on graph but obtained through backward and forward difference method, respectively). Values are expressed as eV. Derivatives are obtained through numerically differentiating the energy values *via* the central difference method.

4 RT-TDDFT analysis

4.1 Vibration [a]

4.1.1 Negative displacement

Table S6: LR-TD-B3LYP vertical excitation energies ($\Delta E^{S_n}(Q(-0.025_{[\mathbf{a}]}))$) for the N3^{4-} [**a**]-distorted structure. Energies are expressed in eV. MO pairs involved and their largest coefficients in the CI expansion according to the LR-TD-DFT formalism (in parenthesis for each pair, as squared values) in the MO basis pairs are reported. h and l stand for HOMO and LUMO, respectively.

S_n	$\Delta E^{S_n}(Q(-0.025_{[\mathbf{a}]}))$	MO pairs
S_{19}	3.095	h-1→l+2 (0.42); h→l+5 (0.05)
S_{20}	3.107	h-7→l+1 (0.05); h-2→l+2 (0.04); h→l+4 (0.38)
S_{21}	3.145	h-3→l (0.18); h-3→l+1 (0.33)
S_{23}	3.187	h-2→l+2 (0.44); h→l+4 (0.03)
S_{24}	3.152	h-16→l+1 (0.02); h-6→l (0.02); h-5→l+1 (0.04); h-2→l+5 (0.01); h-1→l+2 (0.03); h-1→l+4 (0.02); h→l+3 (0.1); h→l+5 (0.28)

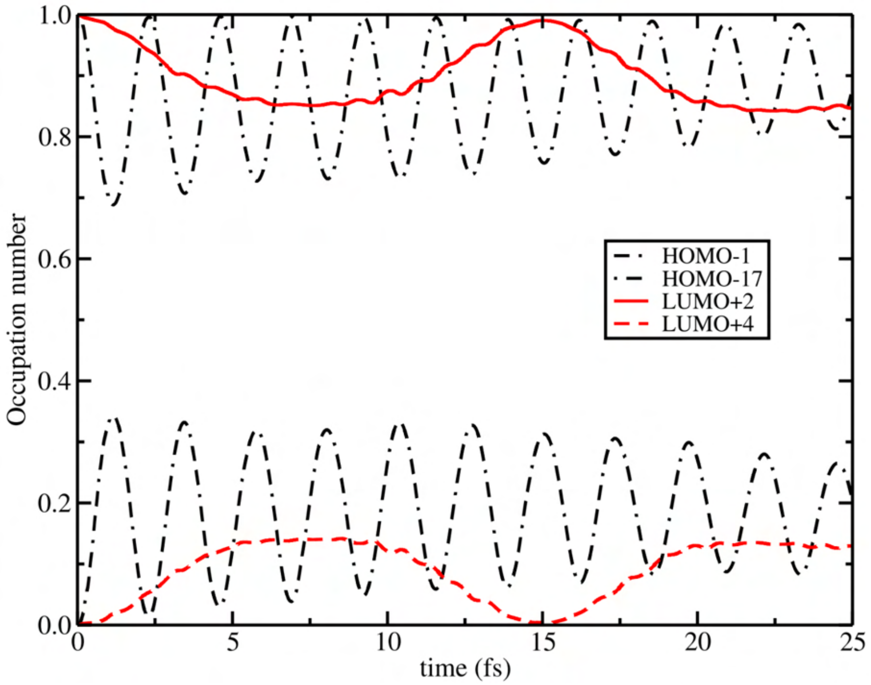

Figure S16: MO occupation number dynamics in $N3^{4-}$ for state $^1MLCT_A$ under negative displacement ($Q_{[\mathbf{a}]} = -0.025$) of [**a**]. Only alpha MO are reported. Individual frontier MO occupation number evolution is reported according to the legend in the graph, where in black and in red are represented the original occupied and virtual MOs for the ground state. Electronic dynamics was prepared according to the MO swap procedure explain in the Computational Methods and Details section, where in this case the HOMO-1 was emptied (dash-dash-dotted black line) and the LUMO+2 (full red line) was populated instead at t=0.

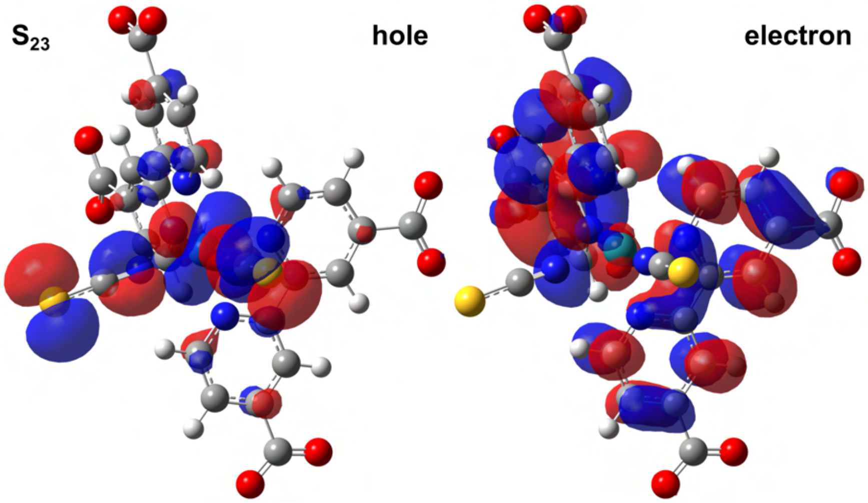

Figure S17: Hole and electron NTOs for the electronic transition towards the adiabatic S_{23} under negative displacement ($Q_{[\mathbf{a}]} = -0.025$) of [**a**]. Isovalue: 0.02.

4.1.2 Positive displacement

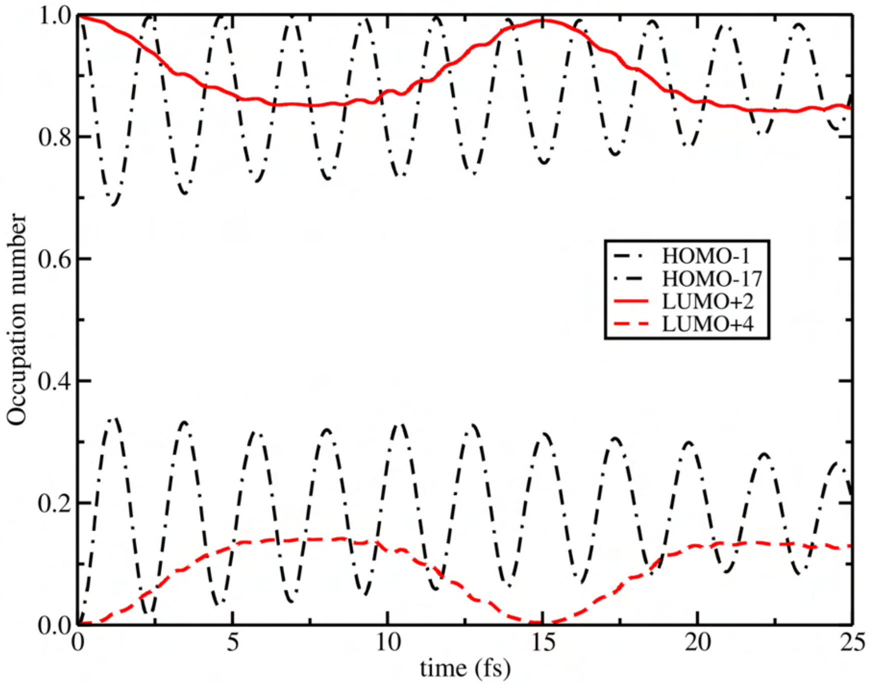

Figure S18: MO occupation number dynamics in $N3^{4-}$ for state $^1MLCT_A$ under positive displacement ($Q_{[\mathbf{a}]} = +0.025$) of [**a**]. Only alpha MO are reported. Individual frontier MO occupation number evolution is reported according to the legend in the graph, where in black and in red are represented the original occupied and virtual MOs for the ground state. Electronic dynamics was prepared according to the MO swap procedure explain in the Computational Methods and Details section, where in this case the HOMO-1 was emptied (dash-dash-dotted black line) and the LUMO+2 (full red line) was populated instead at t=0.

Table S7: LR-TD-B3LYP vertical excitation energies ($\Delta E^{S_n}(Q(+0.025_{[\mathbf{a}]}))$) for the N3^{4-} [**a**]-distorted structure. Energies are expressed in eV. MO pairs involved and their largest coefficients in the CI expansion according to the LR-TD-DFT formalism (in parenthesis for each pair, as squared values) in the MO basis pairs are reported. h and l stand for HOMO and LUMO, respectively.

S_n	$\Delta E^{S_n}(Q(+0.025_{[\mathbf{a}]}))$	MO pairs
S_{19}	3.071	h-1→l+2 (0.42); h→l+5 (0.06)
S_{20}	3.119	h-7→l+1 (0.40); h-2→l+2 (0.08); h→l+4 (0.02)
S_{21}	3.132	h-7→l+1 (0.18); h-2→l+2 (0.38); h-1→l+3 (0.01); h→l+4 (0.03)
S_{23}	3.155	h-4→l (0.17); h-4→l+1 (0.33)
S_{24}	3.166	h-2→l+1 (0.01); h-2→l+5 (0.02); h-1→l+2 (0.04); h-1→l+4 (0.03); h→l+3 (0.02); h→l+5 (0.31)

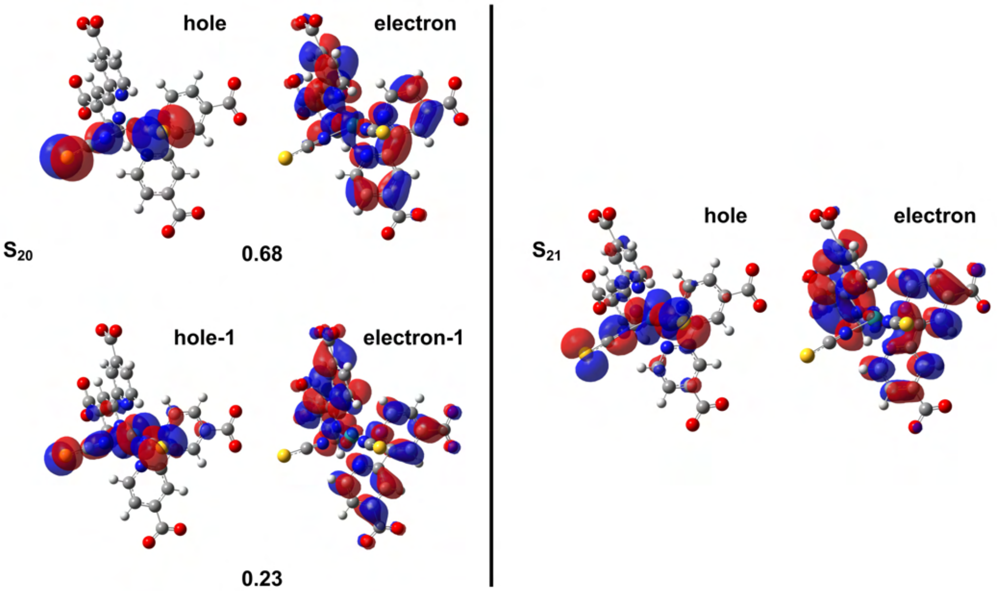

Figure S19: Hole and electron NTOs for the electronic transitions towards the adiabatic S_{20} and S_{21} electronic states under positive displacement ($Q_{[\mathbf{a}]} = +0.025$) of [**a**] and [**b**] ($Q_{[\mathbf{b}]} = +0.025$). Isovalue: 0.02.

4.2 Vibration [b]

Table S8: LR-TD-B3LYP vertical excitation energies ($\Delta E^{S_n}(Q(+0.025_{[\mathbf{b}]}))$) for the $N3^{4-}$ [**b**]-distorted structure. Energies are expressed in eV. MO pairs involved and their largest coefficients in the CI expansion according to the LR-TD-DFT formalism (in parenthesis for each pair, as squared values) in the MO basis pairs are reported. h and l stand for HOMO and LUMO, respectively.

S_n	$\Delta E^{S_n}(Q(+0.025_{[\mathbf{b}]}))$	MO pairs
S_{19}	3.122	h-7→l+1 (0.10); h-1→l+2 (0.33); h→l+4 (0.02); h→l+5 (0.04)
S_{20}	3.143	h-7→l+1 (0.20); h-2→l+2 (0.02); h→l+4 (0.24)
S_{21}	3.191	h-3→l (0.03); h-3→l+1 (0.05); h-2→l+2 (0.35); h→l+4 (0.01); h→l+5 (0.03)
S_{23}	3.197	h-4→l (0.17); h-4→l+1 (0.33)
S_{24}	3.220	h-2→l+2 (0.04); h-2→l+5 (0.01); h-1→l+2 (0.03); h-1→l+4 (0.03); h→l+3 (0.01); h→l+5 (0.29)

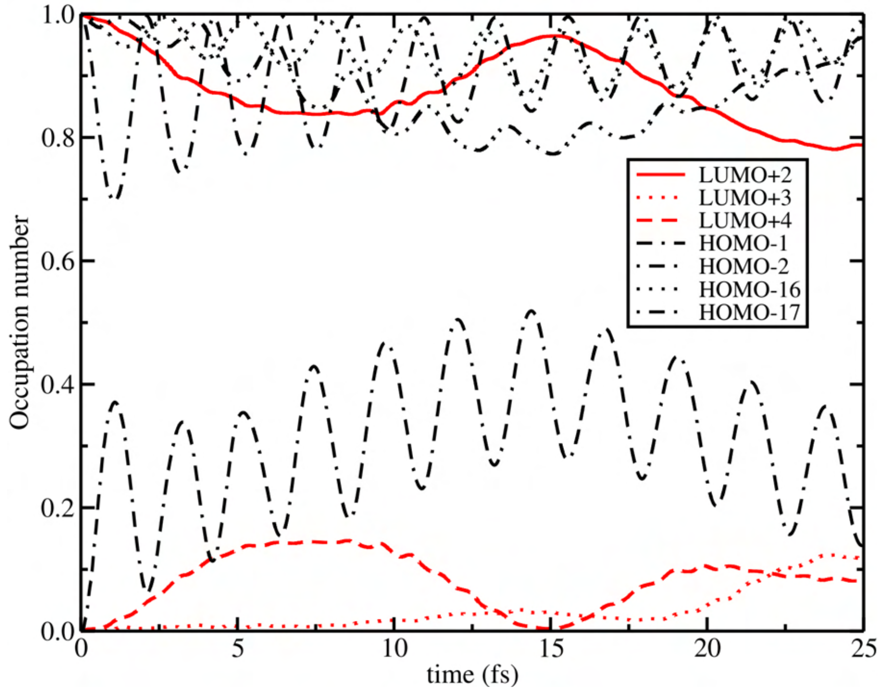

Figure S20: MO occupation number dynamics in $N3^{4-}$ for state $^1MLCT_A$ under positive displacement ($Q_{[\mathbf{b}]} = +0.025$) of [**b**]. Only alpha MO are reported. Individual frontier MO occupation number evolution is reported according to the legend in the graph, where in black and in red are represented the original occupied and virtual MOs for the ground state. Electronic dynamics was prepared according to the MO swap procedure explain in the Computational Methods and Details section, where in this case the HOMO-1 was emptied (dash-dash-dotted black line) and the LUMO+2 (full red line) was populated instead at t=0.

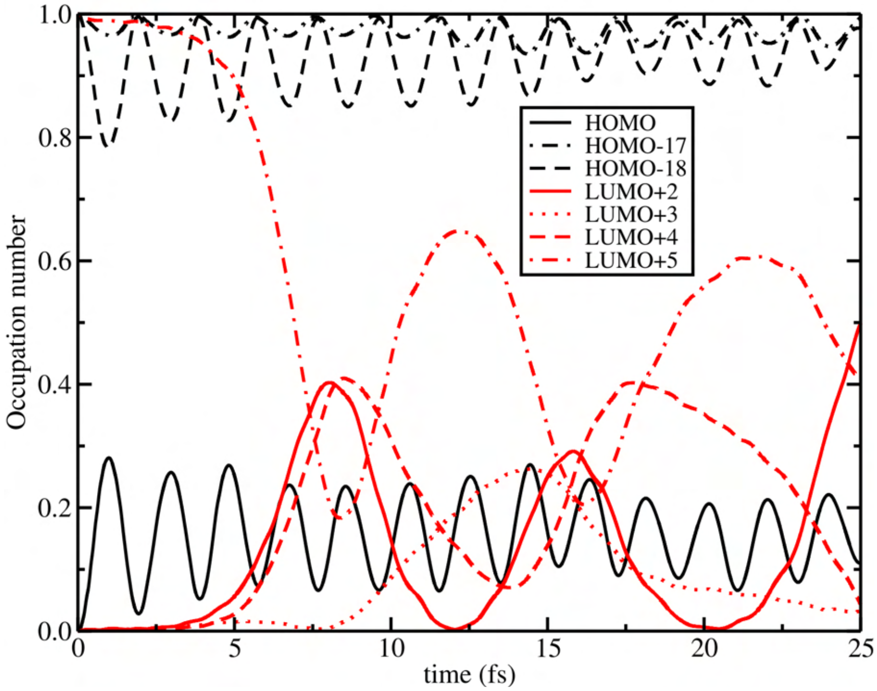

Figure S21: MO occupation number dynamics in $N3^{4-}$ for state $^1MLCT_B$ under positive displacement ($Q_{[\mathbf{b}]} = +0.025$) of [**b**]. Only alpha MO are reported. Individual frontier MO occupation number evolution is reported according to the legend in the graph, where in black and in red are represented the original occupied and virtual MOs for the ground state. Electronic dynamics was prepared according to the MO swap procedure explain in the Computational Methods and Details section, where in this case the HOMO was emptied (full black line) and the LUMO+5 (dot-dashed red line) was populated instead at t=0.

4.3 Vibration [c]

Table S9: LR-TD-B3LYP vertical excitation energies ($\Delta E^{S_n}(Q(+0.18_{[\mathbf{c}]}))$) for the N3^{4-} [**c**]-distorted structure. Energies are expressed in eV. MO pairs involved and their largest coefficients in the CI expansion according to the LR-TD-DFT formalism (in parenthesis for each pair, as squared values) in the MO basis pairs are reported. h and l stand for HOMO and LUMO, respectively.

S$_n$	$\Delta E^{S_n}(Q(+0.18_{[\mathbf{c}]}))$	MO pairs
S_{18}	3.100	h-7→l+1 (0.04); h-2→l+2 (0.01); h-1→l+2 (0.31); h→l+4 (0.10); h→l+5 (0.02)
S_{19}	3.102	h-11→l+1 (0.22); h-11→l+2 (0.24)
S_{20}	3.128	h-7→l+1 (0.35); h-2→l+2 (0.02); h→l+4 (0.11)
S_{21}	3.156	h-4→l (0.22); h-4→l+1 (0.30)
S_{22}	3.168	h-2→l+2 (0.36); h-1→l+2 (0.03); h→l+5 (0.07)
S_{23}	3.187	h-2→l+2 (0.09); h-2→l+5 (0.01); h-1→l+2 (0.03); h-1→l+4 (0.03); h→l+3 (0.02); h→l+4 (0.03); h→l+5 (0.23)

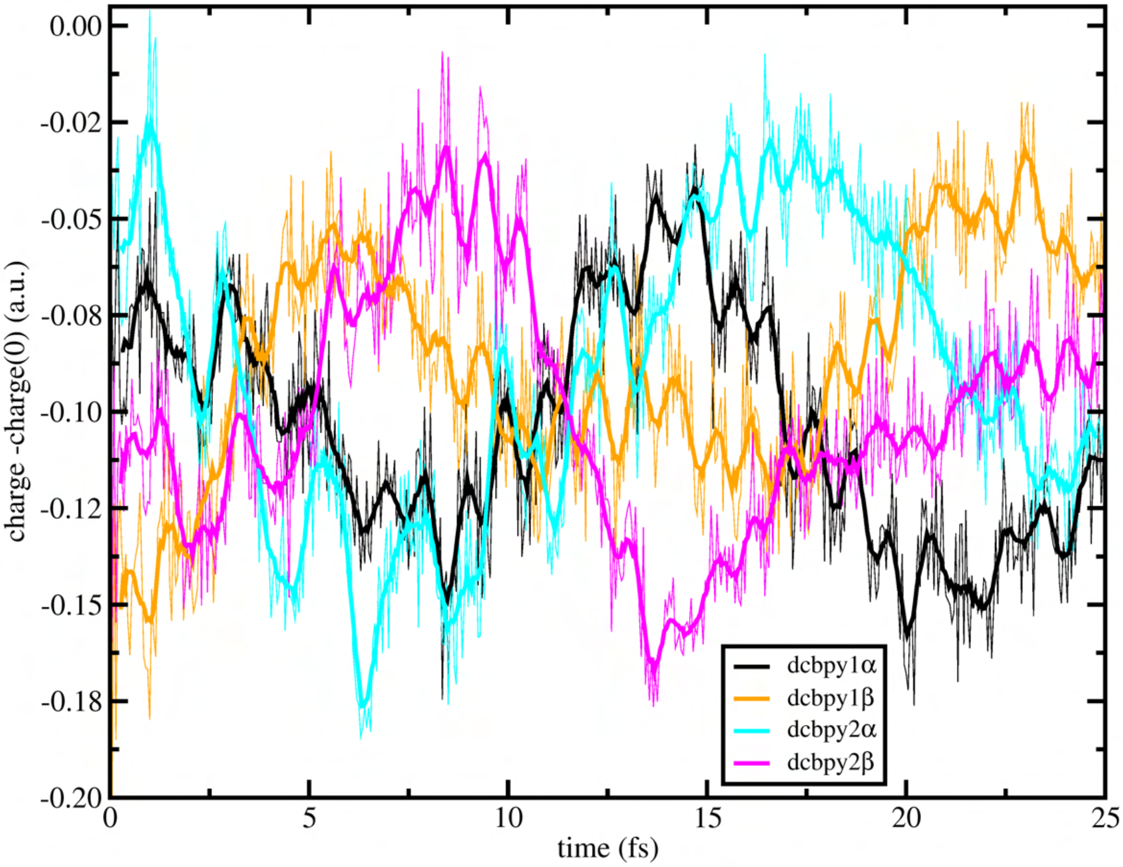

Figure S22: $+0.18_{[c]}$ fragment charges difference dynamics (with respect to the S_0 state) of $^1MLCT_A$ for dcbpy ligands. See Figure 1 for fragment assignment. Mulliken population analysis was performed every 50 as. Line smoothing was done through 10 steps window running averages.

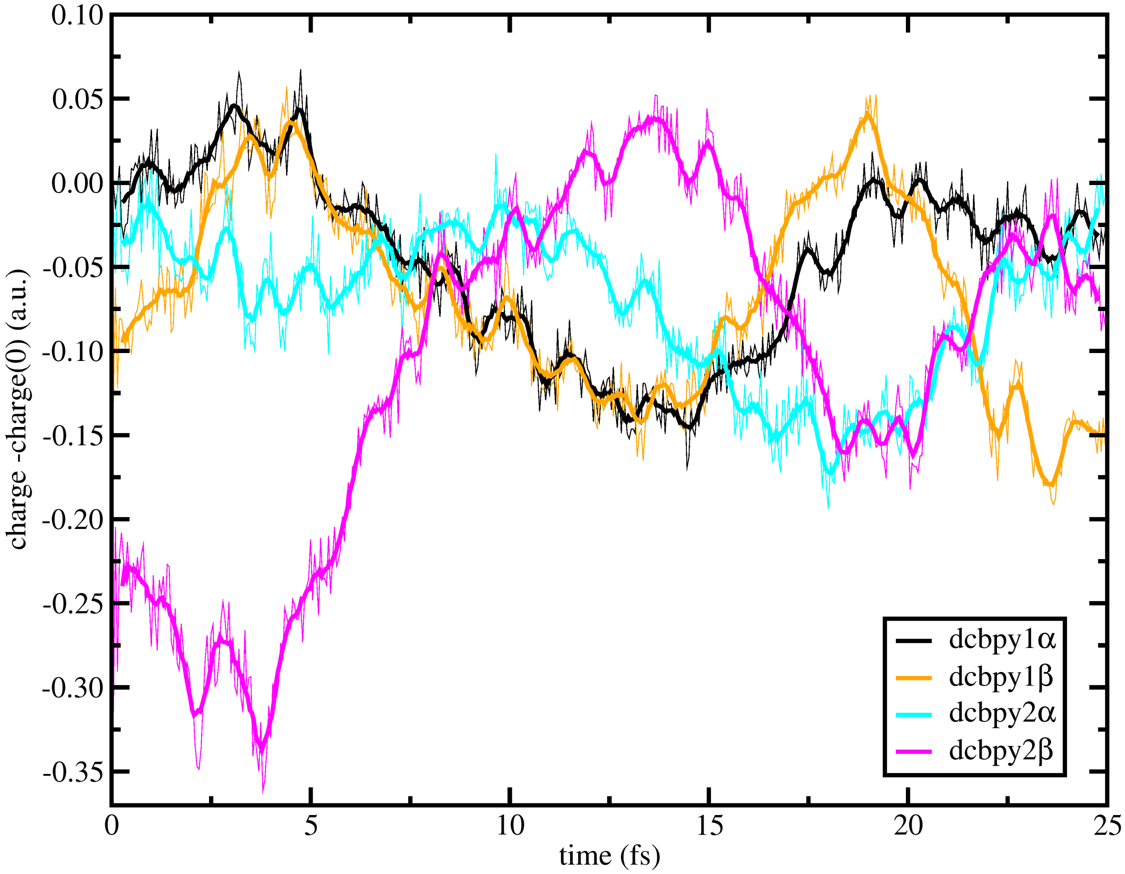

Figure S23: +0.18$_{[c]}$ fragment charges difference dynamics (with respect to the S_0 state) of $^1MLCT_B$ for dcbpy ligands. See Figure 1 in the main text for fragment definition. Mulliken population analysis was performed every 50 as. Line smoothing was done through 10 steps window running averages.

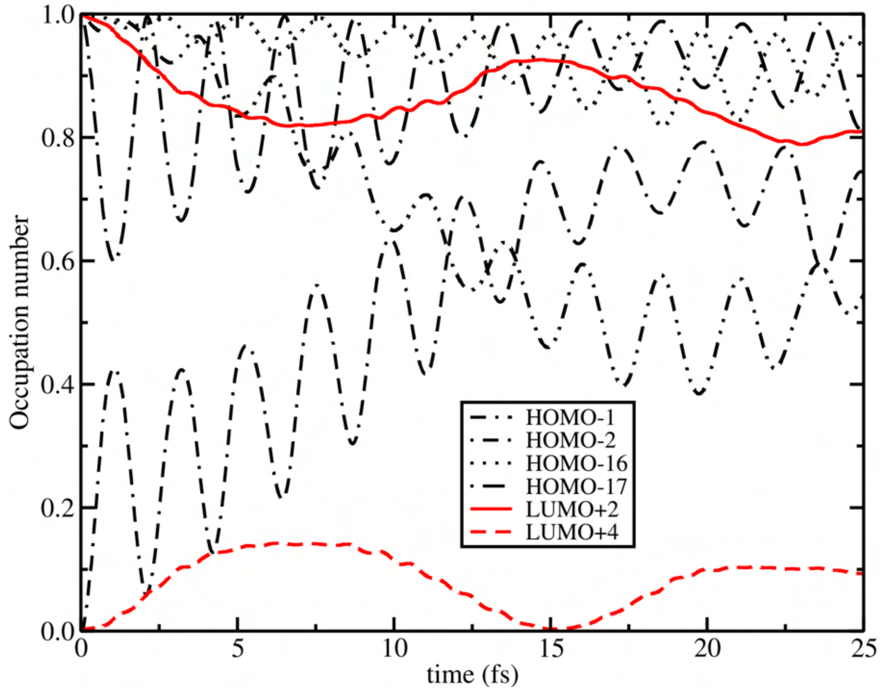

Figure S24: MO occupation number dynamics in $N3^{4-}$ for state $^{1}MLCT_{A}$ under [**c**]-positive displacement ($Q_{[\mathbf{c}]} = +0.18$). Only alpha MO are reported. Individual frontier MO occupation number evolution is reported according to the legend in the graph, where in black and in red are represented the original occupied and virtual MOs for the ground state. Electronic dynamics was prepared according to the MO swap procedure explain in the Computational Methods and Details section, where in this case the HOMO-1 was emptied (dash-dash-dotted black line) and the LUMO+2 (full red line) was populated instead at t=0.

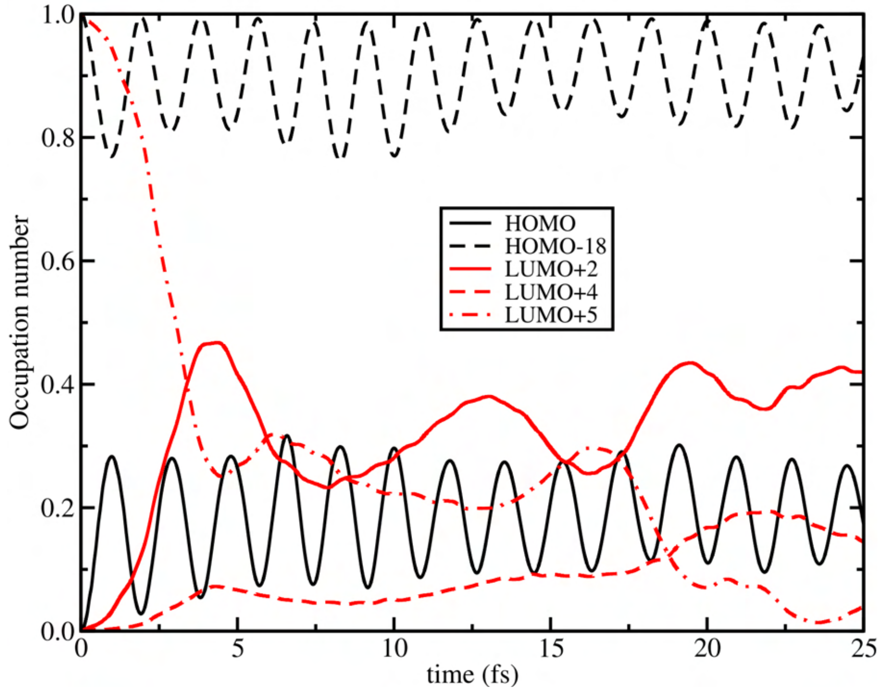

Figure S25: MO occupation number dynamics in $N3^{4-}$ for state $^1MLCT_B$ under [**c**]-positive displacement ($Q_{[\mathbf{c}]} = +0.18$). Only alpha MO are reported. Individual frontier MO occupation number evolution is reported according to the legend in the graph, where in black and in red are represented the original occupied and virtual MOs for the ground state. Electronic dynamics was prepared according to the MO swap procedure explain in the Computational Methods and Details section, where in this case the HOMO was emptied (full black line) and the LUMO+5 (dot-dashed red line) was populated instead at t=0.

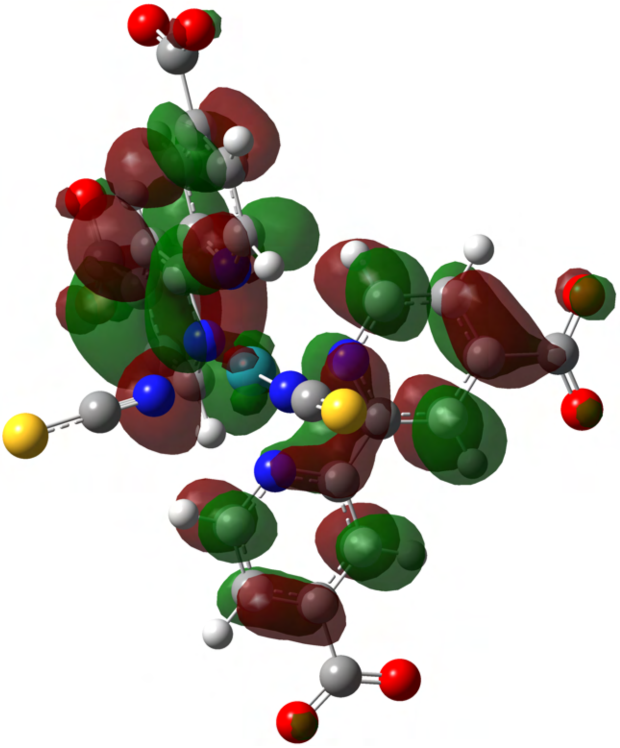

Figure S26: LUMO+2 that is involved into the excitations towards to S_{20}, S_{22} and S_{23} adiabatic electronic states represented for the geometry distorted under the [**c**]-positive displacement ($Q_{[\mathbf{c}]} = +0.18$). Isovalue: 0.02.

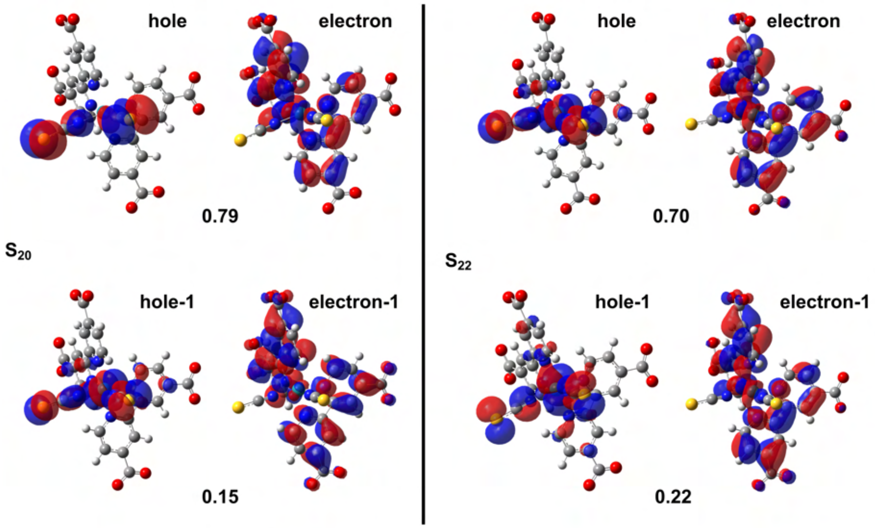

Figure S27: Hole and electron NTOs for the electronic transitions towards the adiabatic S_{20} and S_{22} electronic states under [**c**]-positive displacement ($Q_{[\mathbf{c}]} = +0.18$). Isovalue: 0.02
